# Supplementary material for: A systematic review of the burden of, access to services for and perceptions of patients with overweight and obesity, in humanitarian crisis settings
Source: PLoS One. 2023 Apr 24;18(4):e0282823. doi: 10.1371/journal.pone.0282823 (PMC10124894; doi:10.1371/journal.pone.0282823)
Supplement: S1 Appendix — (DOCX) [file pone.0282823.s002.docx]

## Appendix One: Search Strategies

### Databases

| 94 | Medline | ((exp DISASTERS/ OR (Mass Casualty Event).ti,ab OR exp REFUGEES/ OR exp "WARFARE AND ARMED CONFLICTS"/ OR exp "NATURAL DISASTERS"/ OR exp AVALANCHES/ OR exp EARTHQUAKES/ OR exp FLOODS/ OR exp LANDSLIDES/ OR exp "TIDAL WAVES"/ OR exp TSUNAMIS/ OR exp "CYCLONIC STORMS"/ OR exp DROUGHTS/ OR exp STARVATION/ OR (humanitarian ADJ2 crisis).ti,ab OR (humanitarian ADJ2 crises).ti,ab OR (humanitarian ADJ2 emergenc*).ti,ab OR (humanitarian ADJ2 relief).ti,ab OR (humanitarian ADJ2 response).ti,ab OR (humanitarian ADJ2 agenc*).ti,ab OR (refugee*).ti,ab OR (asylum seeker*).ti,ab OR (evacuee*).ti,ab OR (evacuated*).ti,ab OR (war OR wars OR warfare).ti,ab OR (avalanche*).ti,ab OR (earthquake*).ti,ab OR (flood*).ti,ab OR (landslide*).ti,ab OR (tidal wave*).ti,ab OR (tsunami*).ti,ab OR (cyclon*).ti,ab OR (hurricane*).ti,ab OR (typhoon*).ti,ab OR (drought*).ti,ab OR (famine*).ti,ab OR (starvation*).ti,ab OR (displace* ADJ2 force*).ti,ab OR (displace* ADJ2 population*).ti,ab OR (displace* ADJ2 human).ti,ab OR (displace* ADJ2 internal*).ti,ab OR (displace* ADJ2 person*).ti,ab OR (displace* ADJ2 people*).ti,ab OR (conflict* ADJ2 armed).ti,ab OR (conflict* ADJ2 zone*).ti,ab OR (conflict affected ADJ3 population*).ti,ab OR (conflict affected ADJ3 person*).ti,ab OR (conflict affected ADJ3 people*).ti,ab OR (conflict affected ADJ3 communit*).ti,ab OR (disaster* ADJ3 natural).ti,ab OR (disaster* ADJ3 victim*).ti,ab OR (disaster* ADJ3 plan*).ti,ab OR (disaster* ADJ3 relief*).ti,ab OR (persecution).ti,ab) AND (exp OBESITY/ OR exp OVERWEIGHT/ OR exp "BODY FAT DISTRIBUTION"/ OR (adipos*).ti,ab OR (overweight).ti,ab OR (over weight).ti,ab OR (obes*).ti,ab OR exp "NONCOMMUNICABLE DISEASES"/ OR (NCD).ti,ab OR (non*communicable disease*).ti,ab OR (chronic diseas*).ti,ab OR (chronic condition*).ti,ab OR (long term condition*).ti,ab OR exp "CHRONIC DISEASE"/ OR exp ADIPOSITY/ OR exp "BODY MASS INDEX"/ OR exp "BODY WEIGHT"/ OR exp "WAIST CIRCUMFERENCE"/ OR ("body mass index").ti,ab OR ("waist circumference").ti,ab)) [Humans] |  |
| --- | --- | --- | --- |

| 188 | EMBASE | (((humanitarian ADJ2 crisis).ti,ab OR (humanitarian ADJ2 crises).ti,ab OR (humanitarian ADJ2 emergenc*).ti,ab OR (humanitarian ADJ2 relief).ti,ab OR (humanitarian ADJ2 response).ti,ab OR (humanitarian ADJ2 agenc*).ti,ab OR (refugee*).ti,ab OR (asylum seeker*).ti,ab OR (evacuee*).ti,ab OR (evacuated*).ti,ab OR (war OR wars OR warfare).ti,ab OR (avalanche*).ti,ab OR (earthquake*).ti,ab OR (flood*).ti,ab OR (landslide*).ti,ab OR (tidal wave*).ti,ab OR (tsunami*).ti,ab OR (cyclon*).ti,ab OR (hurricane*).ti,ab OR (typhoon*).ti,ab OR (drought*).ti,ab OR (famine*).ti,ab OR (starvation*).ti,ab OR (displace* ADJ2 force*).ti,ab OR (displace* ADJ2 population*).ti,ab OR (displace* ADJ2 human).ti,ab OR (displace* ADJ2 internal*).ti,ab OR (displace* ADJ2 person*).ti,ab OR (displace* ADJ2 people*).ti,ab OR (conflict* ADJ2 armed).ti,ab OR (conflict* ADJ2 zone*).ti,ab OR (conflict affected ADJ3 population*).ti,ab OR (conflict affected ADJ3 person*).ti,ab OR (conflict affected ADJ3 people*).ti,ab OR (conflict affected ADJ3 communit*).ti,ab OR (disaster* ADJ3 natural).ti,ab OR (disaster* ADJ3 victim*).ti,ab OR (disaster* ADJ3 plan*).ti,ab OR (disaster* ADJ3 relief*).ti,ab OR (persecution).ti,ab OR exp DISASTER/ OR exp REFUGEE/ OR exp WARFARE/ OR exp WAR/ OR exp "NATURAL DISASTER"/ OR exp AVALANCHE/ OR exp EARTHQUAKE/ OR exp FLOODING/ OR exp LANDSLIDE/ OR exp TSUNAMI/ OR exp HURRICANE/ OR exp DROUGHT/ OR exp STARVATION/ OR exp HUNGER/) AND (exp "NONCOMMUNICABLE DISEASES"/ OR (NCD).ti,ab OR (non*communicable disease*).ti,ab OR (chronic diseas*).ti,ab OR (chronic condition*).ti,ab OR (long term condition*).ti,ab OR exp "CHRONIC DISEASE"/ OR exp OBESITY/ OR exp "BODY FAT"/ OR exp "BODY MASS"/ OR exp "BODY WEIGHT"/ OR exp "WAIST CIRCUMFERENCE"/ OR (adipos*).ti,ab OR (overweight).ti,ab OR (over weight).ti,ab OR (obes*).ti,ab OR (body mass index).ti,ab OR (waist circumference).ti,ab)) [Humans] |  |
| --- | --- | --- | --- |

| 79 | PsycINFO | (exp DISASTERS/ OR exp "NATURAL DISASTERS"/ OR exp REFUGEES/ OR exp WAR/ OR exp STARVATION/ OR (humanitarian ADJ2 crisis).ti,ab OR (humanitarian ADJ2 crises).ti,ab OR (humanitarian ADJ2 emergenc*).ti,ab OR (humanitarian ADJ2 relief).ti,ab OR (humanitarian ADJ2 response).ti,ab OR (humanitarian ADJ2 agenc*).ti,ab OR (refugee*).ti,ab OR (asylum seeker*).ti,ab OR (evacuee*).ti,ab OR (evacuated*).ti,ab OR (war OR wars OR warfare).ti,ab OR (avalanche*).ti,ab OR (earthquake*).ti,ab OR (flood*).ti,ab OR (landslide*).ti,ab OR (tidal wave*).ti,ab OR (tsunami*).ti,ab OR (cyclon*).ti,ab OR (hurricane*).ti,ab OR (typhoon*).ti,ab OR (drought*).ti,ab OR (famine*).ti,ab OR (starvation*).ti,ab OR (displace* ADJ2 force*).ti,ab OR (displace* ADJ2 population*).ti,ab OR (displace* ADJ2 human).ti,ab OR (displace* ADJ2 internal*).ti,ab OR (displace* ADJ2 person*).ti,ab OR (displace* ADJ2 people*).ti,ab OR (conflict* ADJ2 armed).ti,ab OR (conflict* ADJ2 zone*).ti,ab OR (conflict affected ADJ3 population*).ti,ab OR (conflict affected ADJ3 person*).ti,ab OR (conflict affected ADJ3 people*).ti,ab OR (conflict affected ADJ3 communit*).ti,ab OR (disaster* ADJ3 natural).ti,ab OR (disaster* ADJ3 victim*).ti,ab OR (disaster* ADJ3 plan*).ti,ab OR (disaster* ADJ3 relief*).ti,ab OR (persecution).ti,ab) AND ((NCD).ti,ab OR (non*communicable disease*).ti,ab OR (chronic diseas*).ti,ab OR (chronic condition*).ti,ab OR (long term condition*).ti,ab OR exp OVERWEIGHT/ OR exp OBESITY/ OR exp "OBESITY (ATTITUDES TOWARD)"/ OR exp "BODY FAT"/ OR exp "BODY WEIGHT"/ OR exp "BODY MASS INDEX"/ OR (adipos*).ti,ab OR (overweight).ti,ab OR (over weight).ti,ab OR (obes*).ti,ab OR (body mass index).ti,ab OR (waist circumference).ti,ab) |  |
| --- | --- | --- | --- |

| 107 | CINAHL | (exp DISASTERS/ OR exp "MASS CASUALTY INCIDENTS"/ OR exp "NATURAL DISASTERS"/ OR exp REFUGEES/ OR exp WAR/ OR exp STARVATION/ OR (humanitarian ADJ2 crisis).ti,ab OR (humanitarian ADJ2 crises).ti,ab OR (humanitarian ADJ2 emergenc*).ti,ab OR (humanitarian ADJ2 relief).ti,ab OR (humanitarian ADJ2 response).ti,ab OR (humanitarian ADJ2 agenc*).ti,ab OR (refugee*).ti,ab OR (asylum seeker*).ti,ab OR (evacuee*).ti,ab OR (evacuated*).ti,ab OR (war OR wars OR warfare).ti,ab OR (avalanche*).ti,ab OR (earthquake*).ti,ab OR (flood*).ti,ab OR (landslide*).ti,ab OR (tidal wave*).ti,ab OR (tsunami*).ti,ab OR (cyclon*).ti,ab OR (hurricane*).ti,ab OR (typhoon*).ti,ab OR (drought*).ti,ab OR (famine*).ti,ab OR (starvation*).ti,ab OR (displace* ADJ2 force*).ti,ab OR (displace* ADJ2 population*).ti,ab OR (displace* ADJ2 human).ti,ab OR (displace* ADJ2 internal*).ti,ab OR (displace* ADJ2 person*).ti,ab OR (displace* ADJ2 people*).ti,ab OR (conflict* ADJ2 armed).ti,ab OR (conflict* ADJ2 zone*).ti,ab OR (conflict affected ADJ3 population*).ti,ab OR (conflict affected ADJ3 person*).ti,ab OR (conflict affected ADJ3 people*).ti,ab OR (conflict affected ADJ3 communit*).ti,ab OR (disaster* ADJ3 natural).ti,ab OR (disaster* ADJ3 victim*).ti,ab OR (disaster* ADJ3 plan*).ti,ab OR (disaster* ADJ3 relief*).ti,ab OR (persecution).ti,ab) AND (exp "NONCOMMUNICABLE DISEASES"/ OR exp "CHRONIC DISEASE"/ OR (NCD).ti,ab OR (non*communicable disease*).ti,ab OR (chronic diseas*).ti,ab OR (chronic condition*).ti,ab OR (long term condition*).ti,ab OR exp OBESITY/ OR exp "ATTITUDE TO OBESITY"/ OR exp "BODY MASS INDEX"/ OR exp "BODY WEIGHT"/ OR exp "WAIST CIRCUMFERENCE"/ OR (adipos*).ti,ab OR (overweight).ti,ab OR (over weight).ti,ab OR (obes*).ti,ab OR (body mass index).ti,ab OR (waist circumference).ti,ab) |
| --- | --- | --- |

PROQUEST - IBSS

| Set# | Searched for | Databases |
| --- | --- | --- |
| S1 | MAINSUBJECT.EXACT(&quot;Refugees&quot;) OR MAINSUBJECT.EXACT(&quot;Landslides &amp; mudslides&quot;)?  OR MAINSUBJECT.EXACT(&quot;Floods&quot;) OR MAINSUBJECT.EXACT(&quot;Tidal waves&quot;) OR?  MAINSUBJECT.EXACT(&quot;Earthquakes&quot;) OR MAINSUBJECT.EXACT(&quot;Disasters&quot;) OR?  MAINSUBJECT.EXACT(&quot;Aftershocks&quot;) OR MAINSUBJECT.EXACT(&quot;Avalanches&quot;) OR?  MAINSUBJECT.EXACT(&quot;Tsunamis&quot;) OR OR MAINSUBJECT.EXACT(&quot;War&quot;) OR?  MAINSUBJECT.EXACT(&quot;Starvation&quot;) OR MAINSUBJECT.EXACT(&quot;Famine&quot;) OR?  MAINSUBJECT.EXACT(&quot;Hunger&quot;) | International Bibliography of the Social Sciences (IBSS) |
| S2 | MAINSUBJECT.EXACT("Refugees") OR MAINSUBJECT.EXACT("Landslides & mudslides") OR MAINSUBJECT.EXACT("Floods") OR MAINSUBJECT.EXACT("Tidal waves") OR MAINSUBJECT.EXACT("Earthquakes") OR MAINSUBJECT.EXACT("Disasters") OR MAINSUBJECT.EXACT("Aftershocks") OR MAINSUBJECT.EXACT("Avalanches") OR MAINSUBJECT.EXACT("Tsunamis") OR OR MAINSUBJECT.EXACT("War") OR MAINSUBJECT.EXACT("Starvation") OR MAINSUBJECT.EXACT("Famine") OR MAINSUBJECT.EXACT("Hunger") | International Bibliography of the Social Sciences (IBSS) |
| S3 | ab(humanitarian work) OR ab(humanitarian worker) OR ab(humanitarian crisis) OR ab(humanitarian aid) OR ab(humanitarian intervention) OR ab(disaster) OR ab(mass casualty events) OR ab(refugee*) OR ab(asylum seek*) OR ab(evacuee*) OR ab(evacuated*) OR ab(internal displaced person) OR ab("conflict affected") OR ab(persecution) OR ab(war) OR ab(wars) OR ab(warfare) OR ab("armed conflict") OR ab(avalanche*) OR ab(earthquake*) | International Bibliography of the Social Sciences (IBSS) |
| S4 | ab(flood*) OR ab(landslide*) OR ab("tidal wave*") OR ab(tsunami*) OR ab("cyclonic storm") OR ab(hurricane*) OR ab(drought*`) OR ab(famine*) OR ab(starvation) | International Bibliography of the Social Sciences (IBSS) |
| S5 | S1 OR S2 OR S3 OR S4 | International Bibliography of the Social Sciences (IBSS)  These databases are searched for part of your query. |
| S6 | MAINSUBJECT.EXACT("Chronic illnesses") OR ab(non-communicable disease*) OR ab("NCD*") OR ab("chronic disease*") OR ab("chronic condition*") OR ab("long term condition*") | International Bibliography of the Social Sciences (IBSS) |
| S7 | OBESITY OR MAINSUBJECT.EXACT("Body composition") OR MAINSUBJECT.EXACT("Obesity") OR MAINSUBJECT.EXACT("Body fat") OR MAINSUBJECT.EXACT("Body mass index") OR MAINSUBJECT.EXACT("Weight") OR MAINSUBJECT.EXACT("Body mass index") OR ab("adipos*") OR ab(overweight) OR ab("over weight") OR ab(obes*) OR ab("body mass index") OR ab("waist circumference") | International Bibliography of the Social Sciences (IBSS) |
| S8 | S6 OR S7 | International Bibliography of the Social Sciences (IBSS)  These databases are searched for part of your query. |
| S9 | S5 AND S8 | International Bibliography of the Social Sciences (IBSS)  These databases are searched for part of your query. |
| S11 | (S5 AND S8) AND stype.exact("Scholarly Journals") | International Bibliography of the Social Sciences (IBSS)  These databases are searched for part of your query. |

Web of Science

|  |  |
| --- | --- |
| **Set** | **Save History / Create AlertOpen Saved History** |
|  | |
| # 3 | #2 AND #1  *Indexes=SCI-EXPANDED, SSCI, A&HCI, CPCI-S, CPCI-SSH, ESCI Timespan=All years* |
|  | |
| # 2 | TOPIC: ((non-communicable disease*) OR ("NCD*") OR ("chronic disease*") OR ("chronic condition*") OR ("long term condition*")) *OR* TOPIC: (adipos*) *OR* TOPIC: ("body fat") *OR* TOPIC: (overweight) *OR* TOPIC: ("over weight") *OR* TOPIC: (obes*) *OR* TOPIC: ("body weight") *OR* TOPIC: ("body mass index") *OR* TOPIC: ("waist circumference")  *Indexes=SCI-EXPANDED, SSCI, A&HCI, CPCI-S, CPCI-SSH, ESCI Timespan=All years* |
|  | |
| # 1 | TOPIC: (((humanitarian work) OR (humanitarian worker) OR (humanitarian crisis) OR (humanitarian aid) OR (humanitarian intervention) OR (disaster) OR (mass casualty event*) OR (refugee*) OR (asylum seek*) OR (evacuee*) OR (evacuated*) OR (internal displaced person) OR ("conflict affected") OR (persecution) OR (war) OR (wars) OR (warfare) OR ("armed conflict") OR (avalanche*) OR (earthquake*) OR (flood*) OR (landslide*) OR ("tidal wave*") OR (tsunami*) OR ("cyclonic storm") OR (hurricane*) OR (drought*) OR (famine*) OR (starvation)))  *Indexes=SCI-EXPANDED, SSCI, A&HCI, CPCI-S, CPCI-SSH, ESCI Timespan=All years* |

COCHRANE

ID Search Hits

#28 MeSH descriptor: [Natural Disasters] explode all trees 52

#29 MeSH descriptor: [Mass Casualty Incidents] explode all trees 21

#30 MeSH descriptor: [Refugees] explode all trees 100

#31 MeSH descriptor: [Warfare and Armed Conflicts] explode all trees 274

#32 MeSH descriptor: [Avalanches] explode all trees 1

#33 MeSH descriptor: [Earthquakes] explode all trees 33

#34 MeSH descriptor: [Floods] explode all trees 3

#35 MeSH descriptor: [Landslides] explode all trees 0

#36 MeSH descriptor: [Tidal Waves] explode all trees 2

#37 MeSH descriptor: [Tsunamis] explode all trees 2

#38 MeSH descriptor: [Cyclonic Storms] explode all trees 6

#39 MeSH descriptor: [Droughts] explode all trees 4

#40 MeSH descriptor: [Starvation] explode all trees 47

#41 MeSH descriptor: [Relief Work] explode all trees 63

#42 {or #28-#41} 545

#43 ((humanitarian work) OR (humanitarian worker) OR (humanitarian crisis) OR (humanitarian aid) OR (humanitarian intervention) OR (disaster) OR (mass casualty event*) OR (refugee*) OR (asylum seek*) OR (evacuee*) OR (evacuated*) OR (internal displaced person) OR ("conflict affected") OR (persecution) OR (war) OR (wars) OR (warfare) OR ("armed conflict") OR (avalanche*) OR (earthquake*) OR (flood*) OR (landslide*) OR ("tidal wave*") OR (tsunami*) OR ("cyclonic storm") OR (hurricane*) OR (drought*) OR (famine*) OR (starvation)):ti,ab,kw 2341

#44 #42 OR #43 2434

#45 MeSH descriptor: [Noncommunicable Diseases] explode all trees 5

#46 MeSH descriptor: [Chronic Disease] explode all trees 12612

#47 MeSH descriptor: [Obesity] explode all trees 12309

#48 MeSH descriptor: [Overweight] explode all trees 14372

#49 MeSH descriptor: [Body Fat Distribution] explode all trees 849

#50 MeSH descriptor: [Adiposity] explode all trees 681

#51 MeSH descriptor: [Body Mass Index] explode all trees 9681

#52 MeSH descriptor: [Body Weight] explode all trees 25151

#53 MeSH descriptor: [Waist Circumference] explode all trees 976

#54 (non-communicable disease*) OR ("NCD*") OR ("chronic disease*") OR ("chronic condition*") OR ("long term condition*") OR ("adipos*") OR ("overweight") OR ("over weight") OR ("obes*") OR ("body mass index") OR ("waist circumference"):ti,ab,kw 65591

#55 #45 OR #46 OR #47 OR #48 OR #49 OR #50 OR #51 OR #52 OR #53 OR #54 78929

#56 #44 AND #55 **159**

### Online

("humanitarian crisis" OR warfare OR war OR “armed conflict” OR "natural disaster" OR "complex humanitarian emergency" OR refugees) AND (Obesity OR BMI OR “Body mass index” OR overweight OR adiposity OR "waist circumference") filetype:pdf

("humanitarian crisis" OR warfare OR war OR “armed conflict” OR "natural disaster" OR "complex humanitarian emergency" OR refugees) AND (Obesity OR BMI OR “Body mass index” OR overweight OR adiposity OR "waist circumference") filetype:pdf site:https://reliefweb.int

("humanitarian crisis" OR warfare OR war OR “armed conflict” OR "natural disaster" OR "complex humanitarian emergency" OR refugees) AND (Obesity OR BMI OR “Body mass index” OR overweight OR adiposity OR "waist circumference") filetype:pdf site:https://[www.unhcr.org](https://www.unhcr.org/)

("humanitarian crisis" OR warfare OR war OR “armed conflict” OR "natural disaster" OR "complex humanitarian emergency" OR refugees) AND (Obesity OR BMI OR “Body mass index” OR overweight OR adiposity OR "waist circumference") filetype:pdf site:[https://](https://who.int)who.int

("humanitarian crisis" OR warfare OR war OR “armed conflict” OR "natural disaster" OR "complex humanitarian emergency" OR refugees) AND (Obesity OR BMI OR “Body mass index” OR overweight OR adiposity OR "waist circumference") filetype:pdf site:[https://](https://who.int)unicef.org

("humanitarian crisis" OR warfare OR war OR “armed conflict” OR "natural disaster" OR "complex humanitarian emergency" OR refugees) AND (Obesity OR BMI OR “Body mass index” OR overweight OR adiposity OR "waist circumference") filetype:pdf site:[https://](https://who.int)msf.org

("humanitarian crisis" OR warfare OR war OR “armed conflict” OR "natural disaster" OR "complex humanitarian emergency" OR refugees) AND (Obesity OR BMI OR “Body mass index” OR overweight OR adiposity OR "waist circumference") filetype:pdf site:[https://](https://who.int)rescue.org

("humanitarian crisis" OR warfare OR war OR “armed conflict” OR "natural disaster" OR "complex humanitarian emergency" OR refugees) AND (Obesity OR BMI OR “Body mass index” OR overweight OR adiposity OR "waist circumference") filetype:pdf site:[https://](https://who.int)icrc.org

("humanitarian crisis" OR warfare OR war OR “armed conflict” OR "natural disaster" OR "complex humanitarian emergency" OR refugees) AND (Obesity OR BMI OR “Body mass index” OR overweight OR adiposity OR "waist circumference") filetype:pdf site:[https://](https://who.int)cdc.gov

("humanitarian crisis" OR warfare OR war OR “armed conflict” OR "natural disaster" OR "complex humanitarian emergency" OR refugees) AND (Obesity OR BMI OR “Body mass index” OR overweight OR adiposity OR "waist circumference") filetype:pdf site:[https://](https://who.int)alnap.org
