# Supplementary material for: A systematic review of the burden of, access to services for and perceptions of patients with overweight and obesity, in humanitarian crisis settings
Source: PLoS One. 2023 Apr 24;18(4):e0282823. doi: 10.1371/journal.pone.0282823 (PMC10124894; doi:10.1371/journal.pone.0282823)
Supplement: S2 Appendix — (DOCX) [file pone.0282823.s003.docx]

**Appendix Two: Studies excluded at full text screening**

| **Author and Year** | **Title** | **How was the study identified?** | **Final in/out reason** |
| --- | --- | --- | --- |
| Arcaya et al. 2014 | Urban sprawl and body mass index among displaced Hurricane Katrina survivors | Database search | Anthropometric measures taken but insufficient data to include in analysis |
| Baraquoni et al. 2020 | It takes time to unravel the ecology of war in Gaza, Palestine: Long-term changes in maternal, newborn and toddlers' heavy metal loads, and infant and toddler developmental milestones in the aftermath of the 2014 military attacks | Database search | Anthropometric measures taken but insufficient data to include in analysis |
| Bell et al. 2019 | Health Risk Behaviors after Disaster Exposure Among Older Adults | Database search | Anthropometric measures taken but insufficient data to include in analysis |
| Berkowitz et al. 2015 | Diabetes risk and mediators among refugees and immigrants: A longitudinal analysis | Database search | Anthropometric measures taken but insufficient data to include in analysis |
| Beyene et al. 2020 | Nutritional status of children aged 0-60 months in two drought-prone areas of Ethiopia | Database search | Anthropometric measures taken but insufficient data to include in analysis |
| Bilukha et al. 2014 | Nutritional Status of Women and Child Refugees from Syria — Jordan, April–May 2014 | Google search | Anthropometric measures taken but insufficient data to include in analysis |
| Bilukha et al. 2020 | Comparison of anthropometric data quality in children aged 6-23 and 24-59 months: lessons from population-representative surveys from humanitarian settings | Database search | Anthropometric measures taken but insufficient data to include in analysis |
| Careyva et al. 2015 | The effect of living in the United States on body mass index in refugee patients | Database search | Anthropometric measures taken but insufficient data to include in analysis |
| Carruth et al. 2020 | Diabetes in a humanitarian crisis: Atypical clinical presentations and challenges to clinical- and community-based management among Somalis in Ethiopia | Database search | Anthropometric measures taken but insufficient data to include in analysis |
| Cherian et al. 2009 | Helicobacter pylori, helminth infections and growth: A cross-sectional study in a high prevalence population | Database search | Anthropometric measures taken but insufficient data to include in analysis |
| Cho et al. 2015 | Characteristics of Body Composition and Muscle Strength of North Korean Refugees during South Korean Stay | Database search | Anthropometric measures taken but insufficient data to include in analysis |
| Chung et al. 2020 | The incidence of diabetes among the non-diabetic residents in Kawauchi village, Fukushima, who experienced evacuation after the 2011 Fukushima Daiichi nuclear power plant disaster | Database search | Anthropometric measures taken but insufficient data to include in analysis |
| Dahab et al. 2020 | Armed conflict as a determinant of children malnourishment: a cross-sectional study in The Sudan | Database search | Anthropometric measures taken but insufficient data to include in analysis |
| Doocy et al. 2017 | Pilot Testing and Implementation of a mHealth tool for Non-communicable Diseases in a Humanitarian Setting | Google search | Anthropometric measures taken but insufficient data to include in analysis |
| El Hatw et al. 2015 | The association of exposure to the 2009 south war with the physical, psychological, and family well-being of Saudi children | Google search | Anthropometric measures taken but insufficient data to include in analysis |
| Fujii et al. 2019 | Circulating microRNAs (miR-126, miR-197, and miR-223) are associated with chronic kidney disease among elderly survivors of the Great East Japan Earthquake | Database search | Anthropometric measures taken but insufficient data to include in analysis |
| Glew et al. 2003 | Effects of displacement resulting from ethnic/religious conflict on the growth and body composition of Fulani children in Northern Nigeria | Database search | Anthropometric measures taken but insufficient data to include in analysis |
| Hammami et al. 2006 | Anthropometric status in Palestinian children living in refugee camps in Lebanon | Database search | Anthropometric measures taken but insufficient data to include in analysis |
| Hashimoto et al. 2017 | Influence of post-disaster evacuation on incidence of metabolic syndrome | Database search | Anthropometric measures taken but insufficient data to include in analysis |
| Hashimoto et al. 2020 | Influence of post-disaster evacuation on incidence of hyperuricemia in residents of Fukushima Prefecture: the Fukushima Health Management Survey | Database search | Anthropometric measures taken but insufficient data to include in analysis |
| Hikichi et al. 2021 | Six-year follow-up study of residential displacement and health outcomes following the 2011 Japan Earthquake and Tsunami | Database search | Anthropometric measures taken but insufficient data to include in analysis |
| Hosseinzadeh Attar et al. 2019 | Assessment of malnutrition and its anthropometric measurement among 0-59 month children aged at Amibara and Awash Fentale districts, afar national regional state of Ethiopia | Database search | Anthropometric measures taken but insufficient data to include in analysis |
| Hu et al. 2016 | Dietary attitudes and behaviours of women in China after the 2008 Wenchuan earthquake in three seismically different zones | Database search | Anthropometric measures taken but insufficient data to include in analysis |
| Isojima et al. 2017 | Prolonged elevated body mass index in preschool children after the Great East Japan Earthquake | Database search | Anthropometric measures taken but insufficient data to include in analysis |
| Jakić et al. 2006 | Secular growth trend in urban children enrolling primary school in the war time | Database search | Anthropometric measures taken but insufficient data to include in analysis |
| Jebril et al. 2020 | Prevalence of type 2 diabetes and its association with added sugar intake in citizens and refugees aged 40 or older in the Gaza strip, Palestine | Database search | Anthropometric measures taken but insufficient data to include in analysis |
| Kikuya et al. 2017 | Alterations in physique among young children after the Great East Japan Earthquake: Results from a nationwide survey | Database search | Anthropometric measures taken but insufficient data to include in analysis |
| Kim et al. 2020 | Prevalence of osteoporosis among North Korean women refugees living in South Korea: A comparative cross-sectional study | Database search | Anthropometric measures taken but insufficient data to include in analysis |
| Knappe et al. 2019 | Impact of an 8-week exercise and sport intervention on post-traumatic stress disorder symptoms, mental health, and physical fitness among male refugees living in a Greek refugee camp | Database search | Anthropometric measures taken but insufficient data to include in analysis |
| Kohrt et al. 2020 | Syndemic effects in complex humanitarian emergencies: A framework for understanding political violence and improving multi-morbidity health outcomes | Database search | Anthropometric measures taken but insufficient data to include in analysis |
| Lane et al. 2019 | Canadian newcomer children's bone health and vitamin D status | Database search | Anthropometric measures taken but insufficient data to include in analysis |
| Markoglou et al. 2005 | Epidemiologic characteristics of hypertension in the civilians of Kosovo after the war | Database search | Anthropometric measures taken but insufficient data to include in analysis |
| Nomura et al. 2016 | Postnuclear disaster evacuation and chronic health in adults in Fukushima, Japan: a long-term retrospective analysis | Database search | Anthropometric measures taken but insufficient data to include in analysis |
| Ono et al. 2018 | Effect of the Fukushima earthquake on weight in early childhood: A retrospective analysis | Database search | Anthropometric measures taken but insufficient data to include in analysis |
| Pak et al. 2010 | The growth status of North Korean refugee children and adolescents from 6 to 19 years of age | Database search | Anthropometric measures taken but insufficient data to include in analysis |
| Panter-Brick et al. 2019 | C-reactive protein, Epstein-Barr virus, and cortisol trajectories in refugee and non-refugee youth: Links with stress, mental health, and cognitive function during a randomized controlled trial | Google search | Anthropometric measures taken but insufficient data to include in analysis |
| Pike et al. 2018 | Low-intensity violence and the social determinants of adolescent health among three East African pastoralist communities | Database search | Anthropometric measures taken but insufficient data to include in analysis |
| Riza et al. 2020 | Determinants of refugee and migrant health status in 10 European countries: The mig-healthcare project | Database search | Anthropometric measures taken but insufficient data to include in analysis |
| Sanoh et al. 2020 | Association between Psychological Factors and Evacuation Status and the Incidence of Cardiovascular Diseases after the Great East Japan Earthquake: A Prospective Study of the Fukushima Health Management Survey | Database search | Anthropometric measures taken but insufficient data to include in analysis |
| Schnake-Mahl et al. 2020 | Effects of gentrification on health status after Hurricane Katrina | Database search | Anthropometric measures taken but insufficient data to include in analysis |
| Schwekendiek 2008 | Determinants of well-being in North Korea: Evidence from the post-famine period | Database search | Anthropometric measures taken but insufficient data to include in analysis |
| Schwekendiek and Pak 2009 | Recent growth of children in the two Koreas: a meta-analysis | Database search | Anthropometric measures taken but insufficient data to include in analysis |
| Shiba et al. 2019 | Long-Term Associations between Disaster Experiences and Cardiometabolic Risk: A Natural Experiment from the 2011 Great East Japan Earthquake and Tsunami | Database search | Anthropometric measures taken but insufficient data to include in analysis |
| Shiba et al. 2020 | Cardiometabolic Profiles and Change in Neighborhood Food and Built Environment Among Older Adults: A Natural Experiment | Database search | Anthropometric measures taken but insufficient data to include in analysis |
| Sun et al. 2013 | Clinical characteristics of hypertension among victims in temporary shield district after Wenchuan earthquake in China | Database search | Anthropometric measures taken but insufficient data to include in analysis |
| Takahashi et al. 2016 | Association between relocation and changes in cardiometabolic risk factors: A longitudinal study in tsunami survivors of the 2011 Great East Japan earthquake | Database search | Anthropometric measures taken but insufficient data to include in analysis |
| Tsubokura et al. 2013 | Changes in metabolic profiles after the Great East Japan Earthquake: a retrospective observational study | Database search | Anthropometric measures taken but insufficient data to include in analysis |
| Watanabe et al. 2019 | Disaster-related trauma and blood pressure among young children: a follow-up study after Great East Japan earthquake | Database search | Anthropometric measures taken but insufficient data to include in analysis |
| Yerkudhov et al. 2020 | Anthropometric characteristics of young adults in areas with different ecological risks in the Aral sea region, Uzbekistan | Database search | Anthropometric measures taken but insufficient data to include in analysis |
| Yokomichi et al. 2018 | Impact of the Great East Japan Earthquake on Body Mass Index, Weight, and Height of Infants and Toddlers: An Infant Survey | Database search | Anthropometric measures taken but insufficient data to include in analysis |
| Adamopoulou and Olivieri 2015 | War and Obesity: The Role of Eating Habits | Google search | Conflict or natural disaster not within date range |
| Barnes et al. 2004 | Health risk and promotion behaviors in refugee populations | Database search | Conflict or natural disaster not within date range |
| Bermingham et al. 1999 | Smoking and lipid cardiovascular risk factors in Vietnamese refugees in Australia | Database search | Conflict or natural disaster not within date range |
| Burns 2004 | Effect of migration on food habits of Somali women living as refugees in Australia | Database search | Conflict or natural disaster not within date range |
| Finer et al. 2016 | Is famine exposure during developmental life in rural Bangladesh associated with a metabolic and epigenetic signature in young adulthood? A historical cohort study | Database search | Conflict or natural disaster not within date range |
| Giallo et al. 2017 | The physical and mental health problems of refugee and migrant fathers: Findings from an Australian population-based study of children and their families | Database search | Conflict or natural disaster not within date range |
| Hou 2010 | Can drought increase total calorie availability? The impact of drought on food consumption and the mitigating effects of a conditional cash transfer program | Database search | Conflict or natural disaster not within date range |
| Kasumova 2011 | Work-related coronary risk factors in refugees and migrants settled in Sumgait City | Database search | Conflict or natural disaster not within date range |
| McEniry et al. 2019 | Displacement due to armed conflict and violence in childhood and adulthood and its effects on older adult health: The case of the middle-income country of Colombia | Database search | Conflict or natural disaster not within date range |
| McEniry et al. 2019 | Early Life Displacement Due to Armed Conflict and Violence, Early Nutrition, and Older Adult Hypertension, Diabetes, and Obesity in the Middle-Income Country of Colombia | Database search | Conflict or natural disaster not within date range |
| Mukeshimana 2001 | Health assessment of Bosnian refugees in Black Hawk County, Iowa | Database search | Conflict or natural disaster not within date range |
| Nelson-Peterman et al. 2015 | Long-Term Refugee Health: Health Behaviors and Outcomes of Cambodian Refugee and Immigrant Women | Database search | Conflict or natural disaster not within date range |
| Peterman et al. 2010 | Relationship between past food deprivation and current dietary practices and weight status among Cambodian refugee women in Lowell, MA | Database search | Conflict or natural disaster not within date range |
| Rossi et al. 2005 | Nutritional status and poverty assessment of vulnerable population groups in Armenia | Database search | Conflict or natural disaster not within date range |
| Todoriki 2004 | Nutrition Transition in Post-War Okinawa Exploring the Link Between Diet, Obesity and Longevity | Google search | Conflict or natural disaster not within date range |
| Van Minh et al. 2019 | The cost of implementing Vietnam’s national plan of action for nutrition for 2017–2020 | Google search | Conflict or natural disaster not within date range |
| Yesilyaprak et al. 2007 | Stress symptoms and nutritional status among survivors of the Marmara region earthquakes in Turkey | Database search | Conflict or natural disaster not within date range |
| AbuKishk et al. 2020 | Anaemia prevalence in children newly registered at UNRWA schools: a cross-sectional study | Google search | Duplicate study |
| Adel et al. 2019 | San Antonio refugees: Their demographics, healthcare profiles, and how to better serve them | Database search | Duplicate study |
| AlKasseh et al. 2013 | Risk factors of gestational diabetes mellitus in the refugee population in Gaza Strip: a case–control study | Google search | Duplicate study |
| Chandra et al. 2019 | Prevalence of chronic disease risk factors in 35- to 44-year-old humanitarian arrivals to New South Wales (NSW), Australia | Database search | Duplicate study |
| Chernet et al. 2019 | Cardiovascular diseases risk factors among recently arrived Eritrean refugees in Switzerland | Google search | Duplicate study |
| Davis et al. 2020 | Prevalence of Overweight and Obesity in US-Bound Refugees: 2009-2017 | Database search | Duplicate study |
| Dookeran et al. 2010 | Chronic Disease and Its Risk Factors Among Refugees and Asylees in Massachusetts, 2001-2005 | Google search | Duplicate study |
| Duffield et al. 1999 | Report on the Nutrition Situation of Refugees and displaced population | Google search | Duplicate study |
| Grijalva-Eternot et al. 2012 | The Double Burden of Obesity and Malnutrition in a Protracted Emergency Setting: A Cross-Sectional Study of Western Sahara | Google search | Duplicate study |
| Heney et al. 2015 | Pediatric Refugees in Rhode Island: Increases in BMI Percentile, Overweight, and Obesity following Resettlement | Google search | Duplicate study |
| Jakic 2005 | Influence of the war events on body weight and height in children enrolling the first grade of elementary school | Database search | Duplicate study |
| Jakic 2006 | Secular growth trend in urban children enrolling primary school in the war time | Database search | Duplicate study |
| Kim and Choi 2020 | Double burden of malnutrition and obesity in children and adolescents from North Korean refugee families | Google search | Duplicate study |
| Kuniyoshi et al. 2019 | Association of Feeding Practice with Childhood Overweight and/or Obesity in Affected Areas before and after the Great East Japan Earthquake | Database search | Duplicate study |
| Mateen et al. 2012 | Medical conditions among Iraqi refugees in Jordan: Data from the United Nations refugee assistance information system | Database search | Duplicate study |
| Mellor et al. 2012 | Aspects of parenting and family functioning associated with obesity in adolescent refugees and migrants from African backgrounds living in Australia | Google search | Duplicate study |
| Mousa et al. 2010 | Hyperglycaemia, hypertension and their risk factors among Palestine refugees served by UNRWA | Google search | Duplicate study |
| Ohira et al. 2019 | External Radiation Dose, Obesity, and Risk of Childhood Thyroid Cancer after the Fukushima Daiichi Nuclear Power Plant Accident: The Fukushima Health Management Survey | Database search | Duplicate study |
| Oltrogge et al. 2020 | Episodes of care in a primary care walk-in clinic at a refugee camp in Germany - a retrospective data analysis | Database search | Duplicate study |
| Ramadan et al. 2016 | Prevalence and correlates of metabolic syndrome in pre-crisis Syria: Call for current relief efforts | Database search | Duplicate study |
| Sastre et al. 2020 | Diet, Physical Activity and Weight-Related Behaviors, Changes and Risks with Newly-Arrived (< 1 Year) Immigrant and Refugee Adolescents (Ages 12-17) | Database search | Duplicate study |
| Sethi et al. 2017 | Community-Based Noncommunicable Disease Care for Syrian Refugees in Lebanon | Google search | Duplicate study |
| Suda et al. 2019 | Medical needs in minamisanriku town after the great east Japan earthquake | Database search | Duplicate study |
| Taylor et al. 2014 | Physical and Mental Health Status of Iraqi Refugees Resettled in the United States | Google search | Duplicate study |
| Watanabe et al. 2019 | Disaster-related trauma and blood pressure among young children: a follow-up study after Great East Japan earthquake | Database search | Duplicate study |
| Caspi et al. 2017 | Food Hardship and Obesity in a Sample of Low-Income Immigrants | Database search | Methods insufficiently described or absent from study |
| Director of Health, UNRWA 2014 | Health conditions in the occupied Palestinian territory, including east Jerusalem, and in the occupied Syrian Golan | Google search | Methods insufficiently described or absent from study |
| Government of Sierra Leone Ministry of Health and Sanitation | Annual Health Sector Performance Report 2016 | Google search | Methods insufficiently described or absent from study |
| Inter-Agency Coordination Lebanon 2017 | Support to Public Institutions in Lebanon under the Lebanon Crisis Response Plan (LCRP 2017-2020) 2017 Results | Google search | Methods insufficiently described or absent from study |
| Njeru et al. 2016 | High Rates of Diabetes Mellitus, Pre-diabetes and Obesity Among Somali Immigrants and Refugees in Minnesota: A Retrospective Chart Review | Database search | Methods insufficiently described or absent from study |
| Nomura et al. 2016 | School restrictions on outdoor activities and weight status in adolescent children after Japan's 2011 Fukushima Nuclear Power Plant disaster: A mid-term to long-term retrospective analysis | Database search | Methods insufficiently described or absent from study |
| Palestinian National Authority Ministry of Health et al. 2005 | The State of Nutrition West Bank and Gaza Strip | Google search | Methods insufficiently described or absent from study |
| Ramadan et al. 2016 | Prevalence and correlates of metabolic syndrome in pre-crisis Syria: call for current relief efforts | Database search | Methods insufficiently described or absent from study |
| Rommel and Ellert 2016 | Health and health-related behavior among people with migrant background in Germany | Database search | Methods insufficiently described or absent from study |
| UNHCR Egypt 2020 | Egypt Response Plan for Refugees and Asylum-Seekers from Sub-Saharan Africa, Iraq & Yemen | Google search | Methods insufficiently described or absent from study |
| Unicef 2010 | The Situation of Palestinian Children in The Occupied Palestinian Territory, Jordan, Syria and Lebanon | Google search | Methods insufficiently described or absent from study |
| US Department of Health and Human Services Centers for Disease Control and Prevention 2003 | CDC Morbidity and Mortality Weekly Report: Health of Resettled Iraqi Refugees — San Diego County, California, October 2007–September 2009 | Google search | Methods insufficiently described or absent from study |
| WHO Regional Office for Europe 2018 | Report on the health of refugees and migrants in the WHO European Region | Google search | Methods insufficiently described or absent from study |
| Acharya et al. 2020 | Exposure to conflict-related violence and nutritional status of children in Iraq | Database search | No anthropometric measures provided in study |
| Al-Odwan et al. 2015 | Women's health Aspect In Humanitarian Missions And Disasters: Jordanian Royal Medical Services Experience | Database search | No anthropometric measures provided in study |
| Andersen et al. 2020 | Health status of refugees newly resettled in Denmark | Database search | No anthropometric measures provided in study |
| Ansbro and et al. 2021 | Clinical outcomes in a primary-level non-communicable disease programme for Syrian refugees and the host population in Jordan: A cohort analysis using routine data | Database search | No anthropometric measures provided in study |
| Arab World for Research and Development (AWRAD) 2020 | Participatory Gender Analysis Report (West Bank & Gaza Strip) | Google search | No anthropometric measures provided in study |
| Ay et al. 2016 | The Perceived Barriers of Access to Health Care Among a Group of Non-camp Syrian Refugees in Jordan | Database search | No anthropometric measures provided in study |
| Aziz et al. 2020 | Diabetic foot and disaster; risk factors for amputation during the Syrian crisis | Database search | No anthropometric measures provided in study |
| Barreau et al. 2017 | Physical, Mental, and Financial Impacts From Drought in Two California Counties, 2015 | Database search | No anthropometric measures provided in study |
| Bertelsen et al. 2018 | Primary Care Screening Methods and Outcomes for Asylum Seekers in New York City | Database search | No anthropometric measures provided in study |
| Biddle et al. 2019 | Health monitoring among asylum seekers and refugees: A state-wide, cross-sectional, population-based study in Germany | Database search | No anthropometric measures provided in study |
| Bischoff et al. 2009 | Health and ill health of asylum seekers in Switzerland: An epidemiological study | Database search | No anthropometric measures provided in study |
| Borgschulte et al. 2018 | Health care provision for refugees in Germany - one-year evaluation of an outpatient clinic in an urban emergency accommodation | Database search | No anthropometric measures provided in study |
| Brackbill et al. 2006 | Surveillance for World Trade Center disaster health effects among survivors of collapsed and damaged buildings | Database search | No anthropometric measures provided in study |
| Burns et al. 2018 | Health status of returning refugees, internally displaced persons, and the host community in a post-conflict district in northern Sri Lanka: a cross-sectional survey | Database search | No anthropometric measures provided in study |
| Burton et al. 2009 | Health of Medicare Advantage plan enrollees at 1 year after Hurricane Katrina | Database search | No anthropometric measures provided in study |
| Bydzovsky et al. 2016 | Experience with migrants on Balkan Route from the Field Hospital on the Slovenian-Croatian Border | Database search | No anthropometric measures provided in study |
| Centers for Disease, Control and Prevention 2006 | Morbidity surveillance after Hurricane Katrina--Arkansas, Louisiana, Mississippi, and Texas, September 2005 | Database search | No anthropometric measures provided in study |
| Centers for Disease, Control and Prevention 2006 | Illness surveillance and rapid needs assessment among Hurricane Katrina evacuees--Colorado, September 1-23, 2005 | Database search | No anthropometric measures provided in study |
| Centers for Disease, Control and Prevention 2010 | Launching a national surveillance system after an earthquake -- Haiti, 2010 | Database search | No anthropometric measures provided in study |
| Chaaban et al. 2019 | Recent changes in welfare indicators among Palestinian refugees in Lebanon: a comparative study of two cross-sectional datasets | Database search | No anthropometric measures provided in study |
| Chan and Griffiths 2009 | Comparison of health needs of older people between affected rural and urban areas after the 2005 Kashmir, Pakistan earthquake | Database search | No anthropometric measures provided in study |
| Chan and Kim 2010 | Characteristics and health outcomes of internally displaced population in unofficial rural self-settled camps after the 2005 Kashmir, Pakistan earthquake | Database search | No anthropometric measures provided in study |
| Chan and Kim 2011 | Chronic health needs immediately after natural disasters in middle-income countries: The case of the 2008 Sichuan, China earthquake | Database search | No anthropometric measures provided in study |
| Chowdhury et al. 2019 | Health Impact of Hurricanes Irma and Maria on St Thomas and St John, US Virgin Islands, 2017-2018 | Database search | No anthropometric measures provided in study |
| Cinaroglu et al. 2020 | EU-28 Country Clusters and Patterns of Disease During the European Refugee Crisis | Database search | No anthropometric measures provided in study |
| Cookson et al. 2008 | Internet-based morbidity and mortality surveillance among Hurricane Katrina evacuees in Georgia | Database search | No anthropometric measures provided in study |
| Daniels et al. 2009 | Access to health services and care-seeking behaviors after the 2007 Ica earthquake in Peru | Database search | No anthropometric measures provided in study |
| Darwish et al. 2020 | Acute primary health care needs of Syrian refugees immediately after arrival to Canada | Database search | No anthropometric measures provided in study |
| Del Pinto et al. 2018 | Health status of Afro-Asian refugees in an Italian urban area: a cross-sectional monocentric study | Database search | No anthropometric measures provided in study |
| Diaz et al. 2015 | Multimorbidity among registered immigrants in Norway: the role of reason for migration and length of stay | Database search | No anthropometric measures provided in study |
| Doocy et al. 2013 | Chronic disease and disability among Iraqi populations displaced in Jordan and Syria | Database search | No anthropometric measures provided in study |
| Dowling et al. 2019 | The association of migration experiences on the self-rated health status among adult humanitarian refugees to Australia: an analysis of a longitudinal cohort study | Database search | No anthropometric measures provided in study |
| Dudova et al. 2016 | Six month follow up in communicable versus non-communicable diseases in an Iraqi refugee camp | Database search | No anthropometric measures provided in study |
| Ekezie et al. 2020 | Self-reported diseases and their associated risk factors among camp-dwelling conflict-affected internally displaced populations in Nigeria | Database search | No anthropometric measures provided in study |
| Elmardi et al. 2020 | Prevalence and determinants of anaemia in women of reproductive age in Sudan: analysis of a cross-sectional household survey | Database search | No anthropometric measures provided in study |
| Food and Agriculture Organization of the United Nations and the World Food Programme 2018 | Monitoring food security in countries with conflict situations: A joint FAO/WFP update for the United Nations Security Council | Google search | No anthropometric measures provided in study |
| Fuhrer et al. 2016 | Morbidity of asylum seekers in a medium-sized German city | Database search | No anthropometric measures provided in study |
| Gammoh 2016 | A preliminary description of medical complaints and medication consumption among 375 Syrian refugees residing in North Jordan | Database search | No anthropometric measures provided in study |
| Gammouh et al. 2015 | Chronic diseases, lack of medications, and depression among Syrian refugees in Jordan, 2013-2014 | Database search | No anthropometric measures provided in study |
| Gerritsen et al. 2006 | Mental and physical health problems of, and the use of healthcare by, Afghan, Iranian and Somali asylum seekers and refugees | Database search | No anthropometric measures provided in study |
| Gerritsen et al. 2006 | Physical and mental health of Afghan, Iranian and Somali asylum seekers and refugees living in the Netherlands | Database search | No anthropometric measures provided in study |
| Gil Cuesta et al. 2020 | Medical Consultations After Typhoon Haiyan in a Field Hospital in the Philippines | Database search | No anthropometric measures provided in study |
| Gottlieb et al. 2020 | Health and Healthcare Utilization among Asylum-Seekers from Berlin's LGBTIQ Shelter: Preliminary Results of a Survey | Database search | No anthropometric measures provided in study |
| Greenough et al. 2008 | Burden of Disease and Health Status Among Hurricane Katrina-Displaced Persons in Shelters: A Population-Based Cluster Sample | Database search | No anthropometric measures provided in study |
| Grievink et al. 2007 | A longitudinal comparative study of the physical and mental health problems of affected residents of the firework disaster Enschede, The Netherlands | Database search | No anthropometric measures provided in study |
| Habib et al. 2011 | Double jeopardy: Assessing the association between internal displacement, housing quality and chronic illness in a low-income neighborhood | Database search | No anthropometric measures provided in study |
| Habib et al. 2014 | Associations between life conditions and multi-morbidity in marginalized populations: the case of Palestinian refugees | Database search | No anthropometric measures provided in study |
| Holman et al. 2008 | Terrorism, acute stress, and cardiovascular health: a 3-year national study following the September 11th attacks | Database search | No anthropometric measures provided in study |
| Hua et al. 2020 | Emergency Department Use Among Assisted Living Residents After Hurricane Irma | Database search | No anthropometric measures provided in study |
| Hussain et al. 2019 | Burden of non-communicable diseases in Iraq after the 2003 war | Database search | No anthropometric measures provided in study |
| Jinnouchi et al. 2020 | Lifestyle factors associated with prevalent and exacerbated musculoskeletal pain after the Great East Japan Earthquake: a cross-sectional study from the Fukushima Health Management Survey | Database search | No anthropometric measures provided in study |
| Jonassen et al. 2018 | Socio-economic status and chronic disease in the West Bank and the Gaza Strip: in and outside refugee camps | Database search | No anthropometric measures provided in study |
| Kampouras et al. 2019 | Child Morbidity and Disease Burden in Refugee Camps in Mainland Greece | Database search | No anthropometric measures provided in study |
| Lafta et al. 2019 | Health and Health Seeking in Mosul During ISIS Control and Liberation: Results From a 40-Cluster Household Survey | Database search | No anthropometric measures provided in study |
| Mahmood et al. 2019 | Nutritional status of preschool and school going children in flood-hit areas of Khyber Pakhtunkhwa Pakistan | Database search | No anthropometric measures provided in study |
| Martinez et al. 2015 | Surveillance for and issues relating to noncommunicable diseases post-Haiyan in Region 8 | Database search | No anthropometric measures provided in study |
| Massad et al. 2012 | Nutritional status of Palestinian preschoolers in the Gaza Strip: a cross-sectional study | Database search | No anthropometric measures provided in study |
| Mateen et al. 2012 | Medical conditions among Iraqi refugees in Jordan: data from the United Nations Refugee Assistance Information System | Database search | No anthropometric measures provided in study |
| McKnight et al. 2020 | Association Between Chronic Medical Conditions and Acute Perinatal Psychiatric Health-Care Encounters Among Migrants: A Population-Based Cohort Study | Database search | No anthropometric measures provided in study |
| Ministry of Health of the Hashemite Kingdom of Jordan et al. 2014 | Joint Rapid Health Facility Capacity and Utilization Assessment (JRHFCUA) | Google search | No anthropometric measures provided in study |
| Mokhtari et al. 2019 | Assessing and comparing nutritional status and related factors among 6-48 months old children born in the damaged rural and urban areas of Varzeghan after the 2012 earthquake | Database search | No anthropometric measures provided in study |
| Monpierre et al. 2016 | Global health of unaccompanied refugee minors in Gironde (France) between 2011 and 2013 | Database search | No anthropometric measures provided in study |
| Oltrogge et al. 2020 | Episodes of care in a primary care walk-in clinic at a refugee camp in Germany â€“ a retrospective data analysis | Database search | No anthropometric measures provided in study |
| Pfortmueller et al. 2013 | Multimorbidity in adult asylum seekers: A first overview | Database search | No anthropometric measures provided in study |
| Ramphal 2018 | Medical and psychosocial needs of the Puerto Rican people after Hurricane Maria | Database search | No anthropometric measures provided in study |
| Redditt et al. 2015 | Health status of newly arrived refugees in Toronto, Ont: Part 2: chronic diseases | Database search | No anthropometric measures provided in study |
| Reed and Barbosa 2017 | Investigating the Refugee Health Disadvantage Among the U.S. Immigrant Population | Database search | No anthropometric measures provided in study |
| Rehr et al. 2018 | Prevalence of non-communicable diseases and access to care among non-camp Syrian refugees in northern Jordan | Database search | No anthropometric measures provided in study |
| Reidy and UNRWA 2019 | How does she cope? Women pushed to new limits in the Gaza strip | Google search | No anthropometric measures provided in study |
| Robards et al. 2020 | Intersectionality: Social marginalisation and self-reported health status in young people | Database search | No anthropometric measures provided in study |
| Rodriguez et al. 2006 | Rapid needs assessment of Hurricane Katrina evacuees-Oklahoma, September 2005 | Database search | No anthropometric measures provided in study |
| Russell et al. 2018 | Addressing the Health and Wellness Needs of Vulnerable Rockaway Residents in the Wake of Hurricane Sandy: Findings From a Health Coaching and Community Health Worker Program | Database search | No anthropometric measures provided in study |
| Salazar et al. 2018 | Health consequences of an armed conflict in Zamboanga, Philippines using a syndromic surveillance database | Database search | No anthropometric measures provided in study |
| Saleh et al. 2018 | Using Mobile Health to Enhance Outcomes of Noncommunicable Diseases Care in Rural Settings and Refugee Camps: Randomized Controlled Trial | Database search | No anthropometric measures provided in study |
| Saleh et al. 2018 | eHealth as a facilitator of equitable access to primary healthcare: the case of caring for non-communicable diseases in rural and refugee settings in Lebanon | Database search | No anthropometric measures provided in study |
| Saulnier et al. 2018 | The Effect of Seasonal Floods on Health: Analysis of Six Years of National Health Data and Flood Maps | Database search | No anthropometric measures provided in study |
| Schafer et al. 2020 | Referrals to secondary care in an outpatient primary care walk-in clinic for refugees in Germany: results from a secondary data analysis based on electronic medical records | Database search | No anthropometric measures provided in study |
| Schnall et al. 2020 | Disaster-Related Shelter Surveillance During the Hurricane Harvey Response - Texas 2017 | Database search | No anthropometric measures provided in study |
| Serre-Delcor et al. 2018 | Health status of asylum Seekers, Spain | Database search | No anthropometric measures provided in study |
| Shih et al. 2020 | Increased medical visits and mortality among adults with cardiovascular diseases in severely affected areas after typhoon morakot | Database search | No anthropometric measures provided in study |
| Soeteman et al. 2007 | Health problems presented to family practices in the Netherlands 1 year before and 1 year after a disaster | Database search | No anthropometric measures provided in study |
| Souhaly et al. 2020 | Factors corresponded to health service utilization of chronic disease management program (Prolanis) patient in health center lateri Ambon city post-earthquake ervina | Database search | No anthropometric measures provided in study |
| Stromme et al. 2020 | Changes in health among Syrian refugees along their migration trajectories from Lebanon to Norway: a prospective cohort study | Database search | No anthropometric measures provided in study |
| Stromme et al. 2020 | Health status and use of medication and their association with migration related exposures among Syrian refugees in Lebanon and Norway: a cross-sectional study | Database search | No anthropometric measures provided in study |
| Strong et al. 2015 | Health status and health needs of older refugees from Syria in Lebanon | Database search | No anthropometric measures provided in study |
| Suda et al. 2019 | Medical needs in minamisanriku town after the great east Japan earthquake | Database search | No anthropometric measures provided in study |
| Trovato et al. 2016 | Dangerous crossing: demographic and clinical features of rescued sea migrants seen in 2014 at an outpatient clinic at Augusta Harbor, Italy | Database search | No anthropometric measures provided in study |
| Truppa et al. 2019 | Utilization of primary health care services among Syrian refugee and Lebanese women targeted by the ICRC program in Lebanon: a cross-sectional study | Database search | No anthropometric measures provided in study |
| van Berlaer et al. 2016 | A refugee camp in the centre of Europe: clinical characteristics of asylum seekers arriving in Brussels | Database search | No anthropometric measures provided in study |
| Vest and Valadez 2006 | Health conditions and risk factors of sheltered persons displaced by Hurricane Katrina | Database search | No anthropometric measures provided in study |
| Wills and Nkowcha 2006 | Health and related factors for Sudanese refugees in Nebraska | Database search | No anthropometric measures provided in study |
| Wu et al. 2015 | A cross-sectional survey on the health status and the health-related quality of life of the elderly after flood disaster in Bazhong city, Sichuan, China | Database search | No anthropometric measures provided in study |
| Zabaneh et al. 2008 | Living and health conditions of Palestinian refugees in an unofficial camp in the Lebanon: A cross-sectional survey | Database search | No anthropometric measures provided in study |
| Emerson et al. 2020 | Maternal mental health symptoms are positively associated with child dietary diversity and meal frequency but not nutritional status in Eastern Democratic Republic of Congo | Database search | No data on overweight or obesity recorded |
| Guglielmi et al. 2020 | ‘We didn’t come here to eat. We came here to save our life’: Health and nutrition challenges facing adolescents in Cox’s Bazar, Bangladesh | Google search | No data on overweight or obesity recorded |
| Hoddinott et al. 2020 | Food transfers, electronic food vouchers and child nutritional status among Rohingya children living in Bangladesh | Database search | No data on overweight or obesity recorded |
| Jayatissa et al. 2006 | Assessment of nutritional status of children under five years of age, pregnant women, and lactating women living in relief camps after the tsunami in Sri Lanka | Database search | No data on overweight or obesity recorded |
| Leidman et al. 2020 | Malnutrition trends in Rohingya children aged 6-59 months residing in informal settlements in Cox's Bazar District, Bangladesh: An analysis of cross-sectional, population-representative surveys | Database search | No data on overweight or obesity recorded |
| Meiqari et al. 2018 | Impact of war on child health in northern Syria: the experience of Médecins Sans Frontieres | Database search | No data on overweight or obesity recorded |
| Olack et al. 2011 | Nutritional status of under-five children living in an informal urban settlement in Nairobi, Kenya | Database search | No data on overweight or obesity recorded |
| Republic of Yemen Ministry of Public Health and Population Central Statistical Organization 2018 | Report on the Nutritional Situation and Mortality Survey Al Jawf Governorate, Yemen | Google search | No data on overweight or obesity recorded |
| Sandell et al. 2017 | Health Status and Anthropometric Changes in Resettled Refugee Children | Database search | No data on overweight or obesity recorded |
| Singh et al. 2006 | Studies on the nutritional status of children aged 0-5 years in a drought-affected desert area of western Rajasthan, India | Database search | No data on overweight or obesity recorded |
| Sun et al. 2013 | The nutritional status of young children and feeding practices two years after the Wenchuan earthquake in the worst-affected areas in China | Database search | No data on overweight or obesity recorded |
| WFP Office of Evaluation 2020 | Evaluation of Cameroon WFP Country Strategic Plan 2018-2020 | Google search | No data on overweight or obesity recorded |
| Whitehall 2008 | Anthropometry and renal size of children suffering under sustained conflict in Sri Lanka | Database search | No data on overweight or obesity recorded |
| Adel et al. 2019 | San Antonio refugees: Their demographics, healthcare profiles, and how to better serve them | Database search | Not possible to differentiate between eligible and ineligible population group |
| Alasagheirin and Clark 2018 | Skeletal growth, body composition, and metabolic risk among North Sudanese immigrant children | Database search | Not possible to differentiate between eligible and ineligible population group |
| Alvarado et al. 2020 | Overweight and obesity in Saharawi women and the relationship with beauty ideals | Database search | Not possible to differentiate between eligible and ineligible population group |
| Asgary et al. 2011 | Communicable and Non-Communicable Diseases Among Recent Immigrants with Implications for Primary care; a Comprehensive Immigrant Health Approach | Database search | Not possible to differentiate between eligible and ineligible population group |
| Ayub et al. 2020 | An Exploration of Chronic Disease Perception, Management, and Barriers to Care in Liberian Refugees Resettled in Charlottesville, Virginia | Database search | Not possible to differentiate between eligible and ineligible population group |
| Babatunde-Sowole et al. 2018 | Exploring the diet and lifestyle changes contributing to weight gain among Australian West African women following migration: A qualitative study | Database search | Not possible to differentiate between eligible and ineligible population group |
| Berge et al. 2019 | Utilizing a Board Game to Measure Family/Parenting Factors and Childhood Obesity Risk | Database search | Not possible to differentiate between eligible and ineligible population group |
| Berkowitz et al. 2016 | Risk of Developing Diabetes Among Refugees and Immigrants: A Longitudinal Analysis | Database search | Not possible to differentiate between eligible and ineligible population group |
| Centers for Disease, Control and Prevention 2006 | Assessment of health-related needs after Hurricanes Katrina and Rita--Orleans and Jefferson Parishes, New Orleans area, Louisiana, October 17-22, 2005 | Database search | Not possible to differentiate between eligible and ineligible population group |
| Centers for Disease, Control and Prevention 2006 | Rapid assessment of health needs and resettlement plans among Hurricane Katrina evacuees--San Antonio, Texas, September 2005 | Database search | Not possible to differentiate between eligible and ineligible population group |
| Chernet et al. 2019 | Cardiovascular diseases risk factors among recently arrived Eritrean refugees in Switzerland | Database search | Not possible to differentiate between eligible and ineligible population group |
| Clarkin 2015 | Methodological issues in the anthropometric assessment of hmong children in the United States | Database search | Not possible to differentiate between eligible and ineligible population group |
| Collins et al. 2017 | Cardiovascular Disease Risk and Prevention Amongst Syrian Refugees: Mixed Methods Study of Médecins Sans Frontières Programme in Jordan | Google search | Not possible to differentiate between eligible and ineligible population group |
| Collins et al. 2017 | Cardiovascular disease risk and prevention amongst Syrian refugees: mixed methods study of Médecins Sans Frontieres programme in Jordan | Database search | Not possible to differentiate between eligible and ineligible population group |
| Cyril et al. 2016 | Relationship between body mass index and family functioning, family communication, family type and parenting style among African migrant parents and children in Victoria, Australia: a parent-child dyad study | Database search | Not possible to differentiate between eligible and ineligible population group |
| Datar et al. 2013 | The impact of natural disasters on child health and investments in rural India | Database search | Not possible to differentiate between eligible and ineligible population group |
| Davis et al. 2020 | Prevalence of Overweight and Obesity in US-Bound Refugees: 2009â€“2017 | Database search | Not possible to differentiate between eligible and ineligible population group |
| Dharod et al. 2013 | Food Insecurity: Its Relationship to Dietary Intake and Body Weight among Somali Refugee Women in the United States | Database search | Not possible to differentiate between eligible and ineligible population group |
| Dorman et al. 2017 | Health Status of North Korean Refugees in Toronto: A Community Based Participatory Research Study | Database search | Not possible to differentiate between eligible and ineligible population group |
| FAFO 2007 | Iraqis in Jordan: Their Number and Characteristics | Google search | Not possible to differentiate between eligible and ineligible population group |
| Fleming et al. 2020 | Food and Me: How adolescents experience nutrition across the world. A Companion Report to The State of the World’s Children 2019 | Google search | Not possible to differentiate between eligible and ineligible population group |
| Greater Manchester Combined Authority 2017 | The Greater Manchester Population Health Plan 2017-2021 | Google search | Not possible to differentiate between eligible and ineligible population group |
| Griffith et al. 2014 | Migration-related influences on obesity among sub-Saharan African migrant adolescents in Melbourne, Australia | Database search | Not possible to differentiate between eligible and ineligible population group |
| Guerin et al. 2007 | Body composition and cardiorespiratory fitness among refugee Somali women living in New Zealand | Database search | Not possible to differentiate between eligible and ineligible population group |
| Hervey et al. 2009 | Overweight among refugee children after arrival in the United States | Database search | Not possible to differentiate between eligible and ineligible population group |
| Kim and Choi 2020 | Double burden of malnutrition and obesity in children and adolescents from North Korean refugee families | Database search | Not possible to differentiate between eligible and ineligible population group |
| Lane et al. 2018 | Chronic health disparities among refugee and immigrant children in Canada | Database search | Not possible to differentiate between eligible and ineligible population group |
| Ma et al. 2020 | Associations between dietary patterns and cardiometabolic risks in japan: A cross-sectional study from the fukushima health management survey, 2011-2015 | Database search | Not possible to differentiate between eligible and ineligible population group |
| Massad et al. 2018 | Metabolic syndrome among refugee women from the west bank, Palestine: A cross-sectional study | Database search | Not possible to differentiate between eligible and ineligible population group |
| Mellor et al. 2012 | Aspects of parenting and family functioning associated with obesity in adolescent refugees and migrants from African backgrounds living in Australia | Database search | Not possible to differentiate between eligible and ineligible population group |
| Ministry of Health FGS et al. 2020 | Somalia micronutrient survey 2019 | Google search | Not possible to differentiate between eligible and ineligible population group |
| Ministry of Health, Jordan 2015 | Ministry of Health, Jordan Public Health Surveillance May - December 2014 | Google search | Not possible to differentiate between eligible and ineligible population group |
| Njeru et al. 2020 | Social networks and obesity among Somali immigrants and refugees | Database search | Not possible to differentiate between eligible and ineligible population group |
| Osiichuk et al. 2020 | Conflict and well-being of civilians: The case of the Russian-Ukrainian hybrid war | Database search | Not possible to differentiate between eligible and ineligible population group |
| Reeve 2020 | Change in health profile of refugees resettling in New Zealand, 1980-2014 | Database search | Not possible to differentiate between eligible and ineligible population group |
| Renzaho et al. 2006 | Obesity and undernutrition in sub-Saharan African immigrant and refugee children in Victoria, Australia | Database search | Not possible to differentiate between eligible and ineligible population group |
| Renzaho et al. 2011 | Prevalence of vitamin D insufficiency and risk factors for type 2 diabetes and cardiovascular disease among African migrant and refugee adults in Melbourne | Database search | Not possible to differentiate between eligible and ineligible population group |
| Renzaho et al. 2012 | Intergenerational differences in food, physical activity, and body size perceptions among African migrants | Database search | Not possible to differentiate between eligible and ineligible population group |
| Saeed et al. 2020 | Profile of risk factors for noncommunicable diseases in major cities of Afghanistan: WHO STEPwise approach | Google search | Not possible to differentiate between eligible and ineligible population group |
| Sastre et al. 2020 | Diet, Physical Activity and Weight-Related Behaviors, Changes and Risks with Newly-Arrived (< 1 Year) Immigrant and Refugee Adolescents (Ages 12â€“17) | Database search | Not possible to differentiate between eligible and ineligible population group |
| Schmied et al. 2020 | Feeding My Child: How mothers experience nutrition across the world. A Companion Report to The State of the World’s Children 2019 | Google search | Not possible to differentiate between eligible and ineligible population group |
| Seagle et al. 2020 | Health screening results of Cubans settling in Texas, USA, 2010-2015: A cross-sectional analysis | Database search | Not possible to differentiate between eligible and ineligible population group |
| Sethi et al. 2017 | Community-Based Noncommunicable Disease Care for Syrian Refugees in Lebanon | Database search | Not possible to differentiate between eligible and ineligible population group |
| Sibai et al. 2020 | Lessons learned in the provision NCD primary care to Syrian refugee and host communities in Lebanon: the need to 'act locally and think globally' | Database search | Not possible to differentiate between eligible and ineligible population group |
| Sotillo and Spizzo 2003 | Anthropometric nutritional evaluation of adults from a disaster survivors community | Database search | Not possible to differentiate between eligible and ineligible population group |
| Sundquist et al. 2010 | Effect of a primary health-care-based controlled trial for cardiorespiratory fitness in refugee women | Database search | Not possible to differentiate between eligible and ineligible population group |
| Tabrizi et al. 2020 | Health Consequences of Lake Urmia in Crisis in the Disaster Area: A Pilot Study | Database search | Not possible to differentiate between eligible and ineligible population group |
| Taylor et al. 2014 | Physical and mental health status of Iraqi refugees resettled in the United States | Database search | Not possible to differentiate between eligible and ineligible population group |
| The Kentucky Office for Refugees 2018 | Kentucky Refugee Health Assessment Report 2018 | Google search | Not possible to differentiate between eligible and ineligible population group |
| UNHCR ACNUR et al. 2019 | 2019 Nutrition Survey Sahrawi Refugee Camps, Tindouf, Algeria | Google search | Not possible to differentiate between eligible and ineligible population group |
| UNHCR et al. 2016 | Interagency Nutrition Surveys amongst Syrian Refugees in Jordan | Google search | Not possible to differentiate between eligible and ineligible population group |
| US Department of Health and Human Services 2003 | Cardiovascular Risk in the Cambodian Community Formative Research from Lowell, Massachusetts | Google search | Not possible to differentiate between eligible and ineligible population group |
| USAID 2018 | Uganda: Nutrition Profile April 2018 | Google search | Not possible to differentiate between eligible and ineligible population group |
| Villena-Esponera et al. 2019 | Food Insecurity and the Double Burden of Malnutrition of Indigenous Refugee Épera Siapidara | Database search | Not possible to differentiate between eligible and ineligible population group |
| Westgard et al. 2020 | Prevalence of Cardiovascular Disease and Risk Factors Among Somali Immigrants and Refugees | Database search | Not possible to differentiate between eligible and ineligible population group |
| Wieland et al. 2012 | Physical activity and nutrition among immigrant and refugee women: a community-based participatory research approach | Database search | Not possible to differentiate between eligible and ineligible population group |
| Yeh et al. 2018 | Immigration Status, Visa Types, and Body Weight Among New Immigrants in the United States | Database search | Not possible to differentiate between eligible and ineligible population group |
| Yun et al. 2012 | High prevalence of chronic non-communicable conditions among adult refugees: implications for practice and policy | Database search | Not possible to differentiate between eligible and ineligible population group |
| Centers for Disease, Control and Prevention 2010 | Health of resettled Iraqi refugees -- San Diego County, California, October 2007-September 2009 | Database search | Publication out of date range |
| Duffield et al. 1999 | Report on the Nutrition Situation of Refugees and Displaced populations | Google search | Publication out of date range |
| Geltman et al. 2010 | Chronic disease and its risk factors among refugees and asylees in Massachusetts, 2001-2005 | Database search | Publication out of date range |
| Kwanbunjan et al. 2006 | Health and nutrition survey of tsunami victims in Phang-Nga Province, Thailand | Database search | Publication out of date range |
| Mousa et al. 2010 | Hyperglycaemia, hypertension and their risk factors among Palestine refugees served by UNRWA | Database search | Publication out of date range |
| Sanlier and Yabanci 2007 | The effects of two earthquakes in the Marmara region of Turkey on the nutritional status of adults | Database search | Publication out of date range |
| UNRWA 2011 | The Annual Report of the Department of Health 2010 | Google search | Publication out of date range |
| Wang et al. 2010 | Survivors of war in the Northern Kosovo (II): baseline clinical and functional assessment and lasting effects on the health of a vulnerable population | Google search | Publication out of date range |
| Yamauchi et al. 2010 | Impact of ethnic conflict on the nutritional status and quality of life of suburban villagers in the Solomon Islands | Database search | Publication out of date range |
| Al-Rousan et al. 2018 | Health needs and priorities of Syrian refugees in camps and urban settings in Jordan: perspectives of refugees and health care providers | Google search | Qualitative study where those with overweight or obesity could not be differentiated from other study participants |
| Al-Rousan et al. 2018 | Health needs and priorities of Syrian refugees in camps and urban settings in Jordan: Perspectives of refugees and health care providers | Database search | Qualitative study where those with overweight or obesity could not be differentiated from other study participants |
| Barnes et al. 2005 | Refugees' perceptions of healthy behaviors | Database search | Qualitative study where those with overweight or obesity could not be differentiated from other study participants |
| Keita et al. 2016 | Applying Concept Mapping Methodology to Identify the Perceptions of Risk and Protective Factors for Childhood Obesity among Southeast Asian Refugees | Database search | Qualitative study where those with overweight or obesity could not be differentiated from other study participants |
| Kim et al. 2007 | Perceptions of Diet and Physical Activity Among California Hmong Adults and Youths | Google search | Qualitative study where those with overweight or obesity could not be differentiated from other study participants |
| Kuo et al. 2016 | Exploratory Study of the Clinical Utility of the Pizzi Healthy Weight Management Assessment (PHWMA) Among Burmese High School Students | Database search | Qualitative study where those with overweight or obesity could not be differentiated from other study participants |
| Renzaho and Dhingra 2017 | Addressing the Needs of Syrian and Iraqi Refugees in the Nepean Blue Mountains Region | Google search | Qualitative study where those with overweight or obesity could not be differentiated from other study participants |
| Rondinelli et al. 2011 | Under- and over-nutrition among refugees in San Diego County, California | Database search | Qualitative study where those with overweight or obesity could not be differentiated from other study participants |
| Wilson et al. 2010 | Towards understanding the new food environment for refugees from the Horn of Africa in Australia | Database search | Qualitative study where those with overweight or obesity could not be differentiated from other study participants |
| World Food Programme 2019 | Social and Behaviour Change Formative Assessment for Anaemia and Obesity Tindouf Refugee Camps, Algeria | Google search | Qualitative study where those with overweight or obesity could not be differentiated from other study participants |
| Abudayya et al 2011 | Diet, nutritional status and school performance among adolescents in Gaza Strip | Google search | Study includes children only |
| Abudayya et al. 2007 | Overweight, stunting, and anemia are public health problems among low socioeconomic groups in school adolescents (12-15 years) in the North Gaza Strip | Database search | Study includes children only |
| AbuKishk et al. 2020 | Anaemia prevalence in children newly registered at UNRWA schools: a cross-sectional study | Database search | Study includes children only |
| Al-Amer et al. 2019 | Accuracy of Body Weight Estimation Among Palestinian Refugee Adolescents Living in Jordan: A Cross-Sectional Study | Database search | Study includes children only |
| Al-Lahham et al. 2019 | Prevalence of underweight, overweight and obesity among Palestinian school-age children and the associated risk factors: a cross sectional study | Google search | Study includes children only |
| Arlinghaus et al. 2020 | Impact of Hurricane Harvey on the Growth of Low Income, Ethnic Minority Adolescents | Database search | Study includes children only |
| Culhane-Pera et al. 2009 | Cardiovascular disease risks in hmong refugees from wat Tham Krabok, Thailand | Database search | Study includes children only |
| Dawson-Hahn et al. 2016 | Growth trajectories of refugee and nonrefugee children in the United States | Database search | Study includes children only |
| Dawson-Hahn et al. 2016 | Comparison of the nutritional status of overseas refugee children with low income children in Washington State | Database search | Study includes children only |
| Drysdale et al. 2020 | Coping through a drought: the association between child nutritional status and household food insecurity in the district of iLembe, South Africa | Database search | Study includes children only |
| El Harake et al. 2018 | Impact of a pilot school-based nutrition intervention on dietary knowledge, attitudes, behavior and nutritional status of Syrian refugee children in the bekaa, Lebanon | Database search | Study includes children only |
| El Kishawi et al. 2016 | Prevalence and associated factors for dual form of malnutrition in mother-child pairs at the same household in the Gaza strip-Palestine | Database search | Study includes children only |
| Ghazi et al. 2014 | Neighbourhood safety and childhood obesity among primary school children in Baghdad City Iraq | Database search | Study includes children only |
| Hamilton et al. 2016 | Psychosocial wellbeing and physical health among Tamil schoolchildren in northern Sri Lanka | Database search | Study includes children only |
| Heney et al. 2015 | Pediatric refugees in Rhode Island: increases in BMI percentile, overweight, and obesity following resettlement | Database search | Study includes children only |
| Hirani et al. 2019 | Medical needs of adolescent refugees resettling in Western Australia | Database search | Study includes children only |
| Hosseinzadeh-Attar et al. 2019 | Assessment of malnutrition and anthropometric measurement among 0-59 months aged children in Amibara and Awash Fentale districts, afar national regional state of Ethiopia | Database search | Study includes children only |
| Idowu et al. 2020 | Determinants of anthropometric characteristics of under-five children in internally displaced persons camps in Abuja municipal area council, Abuja, Nigeria | Database search | Study includes children only |
| Kanao et al. 2009 | Nutritional status correlated with sociodemographic and economic factors among preparatory school-aged children in the Gaza Strip | Database search | Study includes children only |
| Kasaye et al. 2019 | Poor nutrition for under-five children from poor households in Ethiopia: Evidence from 2016 Demographic and Health Survey | Database search | Study includes children only |
| Kawasaki et al. 2020 | Influence of post-disaster evacuation on childhood obesity and hyperlipidemia | Database search | Study includes children only |
| Kuniyoshi et al. 2019 | Association of Feeding Practice with Childhood Overweight and/or Obesity in Affected Areas before and after the Great East Japan Earthquake | Database search | Study includes children only |
| Laukamp et al. 2019 | Health of Syrian unaccompanied asylum seeking adolescents (UASA) at first medical examination in Germany in comparison to UASA from other world regions 11 Medical and Health Sciences 1117 Public Health and Health Services | Database search | Study includes children only |
| Massad et al. 2016 | Double Burden of Undernutrition and Obesity in Palestinian Schoolchildren: A Cross-Sectional Study | Database search | Study includes children only |
| Moriyama et al. 2018 | Obesity in elementary school children after the Great East Japan Earthquake | Database search | Study includes children only |
| O'Donovan et al. 2018 | Nutritional assessment of resettled paediatric refugees in Western Australia | Database search | Study includes children only |
| Ohira et al. 2019 | External radiation dose, obesity, and risk of childhood thyroid cancer after the fukushima daiichi nuclear power plant accident: The fukushima health management survey | Database search | Study includes children only |
| Olson et al. 2017 | Rapid Weight Gain in Pediatric Refugees after US Immigration | Database search | Study includes children only |
| Paul et al. 2012 | Post-Cyclone Sidr nutritional status of women and children in coastal Bangladesh: an empirical study | Database search | Study includes children only |
| Pehlivanturk-Kizilkan et al. 2020 | Nutritional Status of Syrian Refugees in Early Adolescence Living in Turkey | Database search | Study includes children only |
| Pernitez-Agan et al. 2019 | Nutritional profile of Syrian refugee children before resettlement | Database search | Study includes children only |
| Schramm et al. 2016 | Gender and age disparities in adult undernutrition in northern Uganda: high-risk groups not targeted by food aid programmes | Database search | Study includes children only |
| Stellinga-Boelen et al. 2007 | Obesity in asylum seekers' children in The Netherlands - The use of national reference charts | Database search | Study includes children only |
| Trasande et al. 2013 | Associations of World Trade Center exposures with pulmonary and cardiometabolic outcomes among children seeking care for health concerns | Database search | Study includes children only |
| Trasande et al. 2018 | Cardiometabolic profiles of adolescents and young adults exposed to the World Trade Center Disaster | Database search | Study includes children only |
| UNHCR et al. 2019 | Standardised Expanded Nutrition Survey (SENS) Final Report | Google search | Study includes children only |
| Unicef 2016 | Rapid nutritional assessment of under five years old children in internally displaced families in Al-Anbar Governorate 2016 | Google search | Study includes children only |
| Vaid et al. 2019 | Nutritional status of pre-schoolers in the border areas of Jammu | Database search | Study includes children only |
| Walpole et al. 2018 | Cross-sectional growth assessment of children in four refugee camps in Northern Greece | Database search | Study includes children only |
| Yamamura 2016 | Impact of the Fukushima nuclear accident on obesity of children in Japan (2008-2014) | Database search | Study includes children only |
| Yokomichi et al. 2016 | Impact of the great east Japan earthquake on the body mass index of preschool children: A nationwide nursery school survey | Database search | Study includes children only |
| Zheng et al. 2017 | Longitudinal changes in body mass index of children affected by the Great East Japan Earthquake | Database search | Study includes children only |
| Zwi et al. 2017 | Refugee children and their health, development and well-being over the first year of settlement: A longitudinal study | Database search | Study includes children only |
| Abdul-Fatah 2020 | Identify prediabetes risk factors, awareness and dietary pattern among people in Dijil Discrete-Iraq-2019 | Database search | Study population not directly affected by conflict, natural disaster or complex emergencies |
| Doocy et al. 2017 | Guidelines and mHealth to Improve Quality of Hypertension and Type 2 Diabetes Care for Vulnerable Populations in Lebanon: Longitudinal Cohort Study | Database search | Study population not directly affected by conflict, natural disaster or complex emergencies |
| Faramarzi et al. 019 | Association between food insecurity and metabolic syndrome in North West of Iran: Azar Cohort study | Database search | Study population not directly affected by conflict, natural disaster or complex emergencies |
| Farris et al. 2016 | Posttraumatic stress symptoms and body mass index among World Trade Center disaster-exposed smokers: A preliminary examination of the role of anxiety sensitivity | Database search | Study population not directly affected by conflict, natural disaster or complex emergencies |
| Gibson-Helm et al. 2015 | Maternal health and pregnancy outcomes comparing migrant women born in humanitarian and nonhumanitarian source countries: a retrospective, observational study | Database search | Study population not directly affected by conflict, natural disaster or complex emergencies |
| Manjomo et al. 2016 | Managing and monitoring chronic non-communicable diseases in a primary care clinic, Lilongwe, Malawi | Google search | Study population not directly affected by conflict, natural disaster or complex emergencies |
| Saleh et al. 2018 | mHealth use for non-communicable diseases care in primary health: patients' perspective from rural settings and refugee camps | Database search | Study population not directly affected by conflict, natural disaster or complex emergencies |
| Boersma et al. 2021 | Multiple Chronic Conditions Among Veterans and Nonveterans: United States, 2015–2018 | Google search | Study population restricted to military or service personnel |
| Javaheri et al. 2012 | Body Mass Index, waist to hip ratio, and percentage of body fat of the Chemical war survivors in Razavi Khorasan | Google search | Study population restricted to military or service personnel |
| Napier et al. 2017 | Obesity and weight gain among former World Trade Center workers and volunteers | Database search | Study population restricted to military or service personnel |
| Abu Kishk et al. 2019 | Model to improve cardiometabolic risk factors in Palestine refugees with diabetes mellitus attending UNRWA health centers | Database search | Study population selected on the basis of disease type |
| AlKasseh et al. 2013 | Risk factors of gestational diabetes mellitus in the refugee population in Gaza Strip: a case–control study | Google search | Study population selected on the basis of disease type |
| Alkoudsi et al. 2020 | Assessing the effectiveness of a pharmaceutical care service on the quality of life of women with polycystic ovarian syndrome living in war and non-war countries | Database search | Study population selected on the basis of disease type |
| Al-Sharafi and Al-Tahami 2017 | The Effect of War on the Control of Diabetes in Patients with Type 2 Diabetes Mellitus in Yemen: A Cross-Sectional Study | Google search | Study population selected on the basis of disease type |
| Hoshide et al. 2019 | Salt intake and risk of disaster hypertension among evacuees in a shelter after the great East Japan Earthquake | Database search | Study population selected on the basis of disease type |
| Khader et al. 2014 | Diabetes mellitus and treatment outcomes in Palestine refugees in UNRWA primary health care clinics in Jordan | Google search | Study population selected on the basis of disease type |
| Leone et al. 2020 | Dietary Habits of Saharawi Type II Diabetic Women Living in Algerian Refugee Camps: Relationship with Nutritional Status and Glycemic Profile | Database search | Study population selected on the basis of disease type |
| Quast et al. 2019 | Long-term effects of disasters on seniors with diabetes: Evidence from hurricanes Katrina and Rita | Database search | Study population selected on the basis of disease type |
| Abou-Rizk et al. 2020 | Anemia, nutritional status, and breastfeeding practices among mother-child pairs in vulnerable areas of Greater Beirut, Lebanon | Database search | Wrong article type |
| Aghbar et al. 2019 | Prevalence of metabolic syndrome among school children aged 6-18 years in Ein Al-Helwa Palestinian Refugee Camp, Lebanon: a cross-sectional study | Database search | Wrong article type |
| Amirazodi et al. 2018 | Obesity trends and risk factors among refugee children/youth: A scoping review | Database search | Wrong article type |
| Amstutz and Da Costa 2018 | Prevalence of undernutrition and overweight/obesity amongst adult refugees in Geneva, Switzerland | Database search | Wrong article type |
| Arnetz et al. 2017 | Epigenetic risk factors for obesity in trauma-exposed refugees | Database search | Wrong article type |
| Beldowski et al. 2015 | Characterization of the older adult refugee population at Thomas Jefferson university's center for refugee health (CRH) | Database search | Wrong article type |
| Catherine Jen et al. 2018 | Erratum to: Sex Differences and Predictors of Changes in Body Weight and Noncommunicable Diseases in a Random, Newly-Arrived Group of Refugees Followed for Two Years...Erratum to: J Immigrant Minority Health DOI 10.1007/s10903â€‘017â€‘0565â€‘9 | Database search | Wrong article type |
| Cho et al. 2010 | Weight gain and the risk of metabolic syndrome among North Korean refugees living in South Korea - An example of thrifty hypothesis | Database search | Wrong article type |
| Chung and Song 2019 | Study of response to vitamin D replacement in North Korean refugee children and Korean children | Database search | Wrong article type |
| Cope 2012 | Estimating the factors associated with health status and access to care among Iraqis displaced in Jordan and Syria using population assessment data | Database search | Wrong article type |
| Curtis et al. 2018 | Migrant children within Europe: a systematic review of children's perspectives on their health experiences | Database search | Wrong article type |
| Dalmar et al. 2017 | Living in ethnic-enclave neighborhoods may attenuate the negative effect of acculturation on blood pressure in refugees and maintain immigrant health effect | Database search | Wrong article type |
| Di Castelnuovo et al. 2012 | Effect of the L'Aquila earthquake on metabolic syndrome in survivors from the 2009 earthquake in Italy | Database search | Wrong article type |
| Di Castelnuovo et al. 2013 | Metabolic syndrome in survivors from the 2009 earthquake in Italy | Database search | Wrong article type |
| Donker et al. 2005 | Chronic diseases of victims and controls before and after disaster | Database search | Wrong article type |
| Fahd and Abiacar 2019 | Risk factors for diabetes in Palestinians residing in Lebanon: a cross-sectional study | Database search | Wrong article type |
| Fahd and Majed 2018 | Non-communicable disease risk factors in Palestine refugees in Lebanon: A descriptive study | Database search | Wrong article type |
| Fattahi 2019 | Outcomes of implementing the chronic disease self-management program (CDSMP) in the Kurdish community | Database search | Wrong article type |
| Fergus et al. 2012 | Volcanic post traumatic stress and the impact on cardiometabolic health | Database search | Wrong article type |
| Gallegos et al. 2016 | Living well multicultural-does it change behaviours in migrants to reduce the risk of chronic disease? | Database search | Wrong article type |
| Ghattas et al. 2018 | Poverty, food insecurity, and health of Palestinian refugees in Lebanon and recently displaced from Syria to Lebanon: Findings from the 2015 socioeconomic household survey | Database search | Wrong article type |
| Golub 2015 | Longitudinal health outcomes in former refugees | Database search | Wrong article type |
| Grosh 2017 | Adaptability in a Bhutanese refugee community: Navigating integration and the impacts on nutritional health after U.S. resettlement | Database search | Wrong article type |
| Hanna et al. 2015 | Delivering healthcare to the refugee population in Pittsburgh | Database search | Wrong article type |
| Hasegawa et al. 2016 | Emergency Responses and Health Consequences after the Fukushima Accident; Evacuation and Relocation | Database search | Wrong article type |
| Hozawa et al. 2019 | Tohoku medical megabank project community-based cohort study | Database search | Wrong article type |
| Huang 2008 | Immigrant health status, health behavior and health assimilation in the United States | Database search | Wrong article type |
| Ingold and Vatanparast 2014 | Beverage consumption & health of immigrant and refugee children in Canada | Database search | Wrong article type |
| Ishiki et al. 2016 | Housing environment did not affect cognitive and physical status of elderly people displaced from their homes following the great east Japan earthquake: Results from the physical interventional study | Database search | Wrong article type |
| Jimenez et al. 2016 | A nutritional education program development for Saharawi women | Database search | Wrong article type |
| Jonassen et al. 2018 | Socioeconomic status and chronic disease in Palestinians living in and outside refugee camps in the West Bank and the Gaza Strip: An observational study | Database search | Wrong article type |
| Joseph et al. 2012 | Acute lowering of socioeconomic status as a result of hurricane Katrina is associated with later poor health among African Americans | Database search | Wrong article type |
| Jprn 2016 | Intervention study on children's health issues after the Great East Japan Earthquake | Database search | Wrong article type |
| Kayitesi et al. 2015 | Acquisition of cardiovascular disease risk factors among refugees and immigrants: A longitudinal study | Database search | Wrong article type |
| Kearns et al. 2019 | Overweight and obesity among adolescent refugees and migrants arriving in Australia: A review of the literature | Google search | Wrong article type |
| Keidar et al. 2019 | Health of refugees and migrants-where do we stand and what directions should we take? | Database search | Wrong article type |
| Kim et al. 2012 | The comparison of the insulin resistance and the prevalence of metabolic syndrome between North Korean refugees and South Korean | Database search | Wrong article type |
| Kim et al. 2014 | Excess weight gain in nonobese North Korean refugees associated with increased risk of impaired fasting glucose | Database search | Wrong article type |
| Kim et al. 2015 | The association between weight gain and metabolic syndrome among North Korean refugees in South Korea | Database search | Wrong article type |
| Kumar et al. 2011 | The burden of chronic disease among the Bhutanese refugee population at a US Urban clinic | Database search | Wrong article type |
| Lee et al. 2010 | Nutritional status of displaced North Korean children | Database search | Wrong article type |
| Martinez-Lozano et al. 2020 | Impact of hurricanes Irma/Maria on changes in glucose abnormalities and diabetes care | Database search | Wrong article type |
| Médecins Sans Frontières 2015 | Research Protocol - Evaluating the effectiveness and burden of diabetes care in a complex humanitarian emergency setting in Mweso, North Kivu, Democratic Republic of the Congo (DRC), 2015 | Google search | Wrong article type |
| Ming et al. 2012 | Earthquake stress impact on pancreatic alpha-cell and beta-cell function | Database search | Wrong article type |
| Morrison et al. 2018 | When Community Calls, We Collaborate! Community-Based Participatory Research With the Multilanguage Montagnard Refugee Community | Database search | Wrong article type |
| Mosadeghrad et al. 2019 | Impact of food insecurity and malnutrition on the burden of Non-communicable diseases and death in Ethiopia: A situational analysis | Database search | Wrong article type |
| Nagai et al. 2016 | Patterns of body mass index change and its associated lifestyle in evacuees during 3-year after a disaster: Fukushima health management survey | Database search | Wrong article type |
| Nakashima and Yasuda 2015 | Relationship Between The Physical Activity And Obesity Among Children After The Great East Japan Earthquake | Database search | Wrong article type |
| Nishide et al. 2018 | Self-reported rate of eating and prevalence of obesity among children in the great east Japan earthquake affected prefecture | Database search | Wrong article type |
| Ohira et al. 2014 | Changes in metabolic profiles among evacuees after the great east Japan earthquake: The fukushima health management survey | Database search | Wrong article type |
| Ohira et al. 2015 | Overweight people increased in the evacuation zone of fukushima prefecture after the great East Japan earthquake: The Fukushima health management survey | Database search | Wrong article type |
| Pacelli et al. 2016 | Before and after the earthquake of L'Aquila (Central Italy): epidemiological surveillance experiences in the post-seismic period in Italy | Database search | Wrong article type |
| Peterman et al. 2010 | Past food deprivation is related to current dietary practices and weight status in Cambodian refugee women | Database search | Wrong article type |
| Rahim et al. 2019 | Distribution of diseases among followed up patients in Malaysian field hospital during operation starlight-2 | Database search | Wrong article type |
| Rahim et al. 2019 | Providing tertiary healthcare support in humanitarian crisis: An analysis of frequency, distribution and pattern of diseases seen in Malaysian field hospital, cox's bazar, bangladesh | Database search | Wrong article type |
| Rath et al. 2016 | Health needs assessment of children and young adults seeking asylum in Berlin, Germany | Database search | Wrong article type |
| Rawaf 2005 | The health crisis in Iraq | Database search | Wrong article type |
| Renzaho 2004 | Fat, rich and beautiful: Changing socio-cultural paradigms associated with obesity risk, nutritional status and refugee children from sub-Saharan Africa | Database search | Wrong article type |
| Rhodes and Percac-Lima 2015 | Development of obesity and related diseases in a prospective longitudinal cohort of African refugees compared to matched regional controls | Database search | Wrong article type |
| Roozbeh et al. 2018 | Afghan refugees and immigrants health status in Iran: A systematic review | Database search | Wrong article type |
| Rosenthal 2018 | Immigration and Acculturation: Impact on Health and Well-Being of Immigrants | Database search | Wrong article type |
| Ruby et al. 2015 | The effectiveness of interventions for non-communicable diseases in humanitarian crises: A systematic Review | Database search | Wrong article type |
| Sabatinelli et al. 2010 | Health of Palestine refugees in the Eastern Mediterranean: Determinants and challenges | Database search | Wrong article type |
| Samad et al. 2019 | Field hospital mission for refugees-the ministry of health Malaysia experience | Database search | Wrong article type |
| Schramm et al. 2013 | Nutritional status among adults in a post-conflict area, northern Uganda: Are humanitarian assistance programmes creating disparities in health? | Database search | Wrong article type |
| Spiroski and Nikovska 2020 | Prevalence of malnutrition among refugee children traveling on Balkan Route | Database search | Wrong article type |
| Stender et al. 2012 | Profile of a healthy lifestyle clinic in Orleans Parish post-Katrina | Database search | Wrong article type |
| Suneja et al. 2018 | Burden and Management of Noncommunicable Diseases After Earthquakes and Tsunamis | Database search | Wrong article type |
| Takahashi et al. 2015 | Impact of a major natural disaster on longitudinal changes in cardiovascular risk factors in the general population | Database search | Wrong article type |
| Takahashi et al. 2015 | Evacuation after the fukushima daiichi nuclear power plant accident as a cause of liver dysfunction: The fukushima health management survey | Database search | Wrong article type |
| Tsubokura et al. 2014 | The immediate physical and mental health crisis in residents proximal to the evacuation zone after Japan's nuclear disaster: an observational pilot study | Database search | Wrong article type |
| UNICEF 2019 | The State Of The World’s Children 2019: Growing Well In A Changing World Children, Food And Nutrition | Google search | Wrong article type |
| Uscher-Pines 2008 | The physical health impacts of post-disaster displacement: A study of the older adult victims of Hurricane Katrina | Database search | Wrong article type |
| WHO Regional Office for Europe 2019 | Prevention and control of noncommunicable diseases in refugees and migrants Technical guidance | Google search | Wrong article type |
| World Food Programme 2020 | Algeria: An Evaluation Of WFP’s Interim Country Strategic Plan (2019 – 2022) | Google search | Wrong article type |
| Yokomichi et al. 2017 | Impact of the great east Japan earthquake on the body mass index of milk-fed infants and toddlers: A nationwide infant survey | Database search | Wrong article type |
