# Supplementary material for: A systematic review of the burden of, access to services for and perceptions of patients with overweight and obesity, in humanitarian crisis settings
Source: PLoS One. 2023 Apr 24;18(4):e0282823. doi: 10.1371/journal.pone.0282823 (PMC10124894; doi:10.1371/journal.pone.0282823)
Supplement: S3 Appendix — (DOCX) [file pone.0282823.s004.docx]

## Appendix three - prevalence ranges in subgroups of interest

Supplementary Table showing the range of prevalence estimates for overweight, obesity and overweight and obesity combined, organised by pre-specified subgroups of interest

| **subgroup** | **category** | **Range of prevalence overweight** | **Range of prevalence obesity** | **Range of prevalence overweight and obesity** |
| --- | --- | --- | --- | --- |
| Setting | HIC | 9.3% [[1]](https://paperpile.com/c/QLrn6N/kPSjv) to 53% [[2]](https://paperpile.com/c/QLrn6N/QqZV) | 0.5% [[3]](https://paperpile.com/c/QLrn6N/oaPAh) to 44.1% [[4]](https://paperpile.com/c/QLrn6N/LSArl) | 15.9% [[3]](https://paperpile.com/c/QLrn6N/oaPAh) to 81.6% [[5]](https://paperpile.com/c/QLrn6N/3u4rR) |
|  | LMIC | 6% [[6]](https://paperpile.com/c/QLrn6N/XmFCv) to 45.7% [[7]](https://paperpile.com/c/QLrn6N/fZdjU) | 0% [[8]](https://paperpile.com/c/QLrn6N/BhxeZ) to 52.7% [[9]](https://paperpile.com/c/QLrn6N/trR6R) | 6.4% [[6]](https://paperpile.com/c/QLrn6N/XmFCv) to 82.8% [[9]](https://paperpile.com/c/QLrn6N/trR6R) |
| Exposure | Conflict | 6% [[6]](https://paperpile.com/c/QLrn6N/XmFCv) to 53% [[2]](https://paperpile.com/c/QLrn6N/QqZV) | 0% [[8]](https://paperpile.com/c/QLrn6N/BhxeZ) to 52.7% [[9]](https://paperpile.com/c/QLrn6N/trR6R) | 6.4% [[6]](https://paperpile.com/c/QLrn6N/XmFCv) to 82.8% [[9]](https://paperpile.com/c/QLrn6N/trR6R) |
|  | Long-standing refugee situation | 12.4% [[10]](https://paperpile.com/c/QLrn6N/NuW2f) to 42% [[11]](https://paperpile.com/c/QLrn6N/JUzVA) | 1.7% [[10]](https://paperpile.com/c/QLrn6N/NuW2f) to 35.7% [[12]](https://paperpile.com/c/QLrn6N/ztGgi) | 14.1% [[10]](https://paperpile.com/c/QLrn6N/NuW2f) to 64.8% [[13,14]](https://paperpile.com/c/QLrn6N/4bnki+ebftD) |
|  | Natural Disaster | 23.2% [[15]](https://paperpile.com/c/QLrn6N/hlYXs) to 45.7% [[7]](https://paperpile.com/c/QLrn6N/fZdjU) | 3.4% [[16]](https://paperpile.com/c/QLrn6N/oZtmh) to 33.2% [[17]](https://paperpile.com/c/QLrn6N/ECPWl) | 26.4% [[18]](https://paperpile.com/c/QLrn6N/tdZ32) to 81.6% [[5]](https://paperpile.com/c/QLrn6N/3u4rR) |
|  | Mixed | 9.3% [[1]](https://paperpile.com/c/QLrn6N/kPSjv) to 32.7% [[19,20]](https://paperpile.com/c/QLrn6N/cVOQt+PwRjD) | 10% [[21]](https://paperpile.com/c/QLrn6N/ARMM7) to 20.3% [[22]](https://paperpile.com/c/QLrn6N/cQvOZ) | 40.2% [[21]](https://paperpile.com/c/QLrn6N/ARMM7) to 47.7% [[19,20]](https://paperpile.com/c/QLrn6N/cVOQt+PwRjD) |
| Internal ROB | Low | 6% [[6]](https://paperpile.com/c/QLrn6N/XmFCv) to 45.7% [[7]](https://paperpile.com/c/QLrn6N/fZdjU) | 0.4% [[6]](https://paperpile.com/c/QLrn6N/XmFCv) to 52.7% [[9]](https://paperpile.com/c/QLrn6N/trR6R) | 6.4% [[6]](https://paperpile.com/c/QLrn6N/XmFCv) to 82.8% [[9]](https://paperpile.com/c/QLrn6N/trR6R) |
|  | Moderate | 25.8% [[4]](https://paperpile.com/c/QLrn6N/LSArl) to 32.7% [[19,20]](https://paperpile.com/c/QLrn6N/cVOQt+PwRjD) | 3.4% [[16]](https://paperpile.com/c/QLrn6N/oZtmh) to 44.1% [[4]](https://paperpile.com/c/QLrn6N/LSArl) | 26.4% [[18]](https://paperpile.com/c/QLrn6N/tdZ32) to 81.6% [[5]](https://paperpile.com/c/QLrn6N/3u4rR) |
|  | High | 22.1% [[8]](https://paperpile.com/c/QLrn6N/BhxeZ) to 53% [[2]](https://paperpile.com/c/QLrn6N/QqZV) | 0% [[8]](https://paperpile.com/c/QLrn6N/BhxeZ) to 27.1% [[15]](https://paperpile.com/c/QLrn6N/hlYXs) | 17.2% [[23]](https://paperpile.com/c/QLrn6N/xb3ro) to 80%[[2]](https://paperpile.com/c/QLrn6N/QqZV) |
| Displaced | Yes | 6% [[6]](https://paperpile.com/c/QLrn6N/XmFCv) to 53% [[2]](https://paperpile.com/c/QLrn6N/QqZV) | 0.4% [[6]](https://paperpile.com/c/QLrn6N/XmFCv) to 52.7% [[9]](https://paperpile.com/c/QLrn6N/trR6R) | 6.4% [[6]](https://paperpile.com/c/QLrn6N/XmFCv) to 82.8% [[9]](https://paperpile.com/c/QLrn6N/trR6R) |
|  | No | 12.4% [[10]](https://paperpile.com/c/QLrn6N/NuW2f) to 35.7 % [[24]](https://paperpile.com/c/QLrn6N/kxNWj) | 0% [[8]](https://paperpile.com/c/QLrn6N/BhxeZ) to 35.3% [[25]](https://paperpile.com/c/QLrn6N/A7TW6) | 14.1% [[10]](https://paperpile.com/c/QLrn6N/NuW2f) to 57.1% [[24]](https://paperpile.com/c/QLrn6N/kxNWj) |
|  | Mixed | 29.4% [[16]](https://paperpile.com/c/QLrn6N/oZtmh) to 45.7% [[7]](https://paperpile.com/c/QLrn6N/fZdjU) | 3.4% [[16]](https://paperpile.com/c/QLrn6N/oZtmh) to 32.7% [[26]](https://paperpile.com/c/QLrn6N/Hxuka) | 17.2% [[23]](https://paperpile.com/c/QLrn6N/xb3ro) to 81.6% [[5]](https://paperpile.com/c/QLrn6N/3u4rR) |

**References**

1. [Kortas AZ, Polenz J, von Hayek J, Rüdiger S, Rottbauer W, Storr U, et al. Screening for infectious diseases among asylum seekers newly arrived in Germany in 2015: a systematic single-centre analysis. Public Health. 2017;153: 1–8.](http://paperpile.com/b/QLrn6N/kPSjv)

2. [Drummond PD, Mizan A, Burgoyne A, Wright B. Knowledge of cardiovascular risk factors in West African refugee women living in Western Australia. J Immigr Minor Health. 2011;13: 140–148.](http://paperpile.com/b/QLrn6N/QqZV)

3. [Modesti PA, Scali E, Marzotti I, Ulivi N, Boddi M, Galanti G, et al. Blood pressure and fasting glucose changes in male migrants waiting for an asylum decision in Italy. A pilot study. Int J Cardiol. 2020;309: 110–114.](http://paperpile.com/b/QLrn6N/oaPAh)

4. [Maldari T, Elsley N, Rahim RA. The health status of newly arrived Syrian refugees at the Refugee Health Service, South Australia, 2016. Aust J Gen Pract. 2019;48: 480–486.](http://paperpile.com/b/QLrn6N/LSArl)

5. [Takahashi S, Yonekura Y, Sasaki R, Yokoyama Y, Tanno K, Sakata K, et al. Weight Gain in Survivors Living in Temporary Housing in the Tsunami-Stricken Area during the Recovery Phase following the Great East Japan Earthquake and Tsunami. PLoS One. 2016;11: e0166817.](http://paperpile.com/b/QLrn6N/3u4rR)

6. [Singh KP, Bhoopathy SV, Worth H, Seale H, Richmond RL. Nutrition among men and household food security in an internally displaced persons camp in Kenya. Public Health Nutr. 2016;19: 723–731.](http://paperpile.com/b/QLrn6N/XmFCv)

7. [Furusawa T, Furusawa H, Eddie R, Tuni M, Pitakaka F, Aswani S. Communicable and non-communicable diseases in the Solomon Islands villages during recovery from a massive earthquake in April 2007. N Z Med J. 2011;124: 17–28.](http://paperpile.com/b/QLrn6N/fZdjU)

8. [Al-Duais MA, Al-Awthan YS. Association between qat chewing and dyslipidaemia among young males. J Taibah Univ Med Sci. 2019;14: 538–546.](http://paperpile.com/b/QLrn6N/BhxeZ)

9. [Ratnayake R, Rawashdeh F, AbuAlRub R, Al-Ali N, Fawad M, Bani Hani M, et al. Access to Care and Prevalence of Hypertension and Diabetes Among Syrian Refugees in Northern Jordan. JAMA Netw Open. 2020;3: e2021678.](http://paperpile.com/b/QLrn6N/trR6R)

10. [Bayyari WD, Henry LJ, Jones C. Dieting behaviours, obesity and predictors of dieting among female college students at Palestinian universities. Eastern Mediterranean Health Journal. 2013. pp. 30–36. doi:](http://paperpile.com/b/QLrn6N/NuW2f)[10.26719/2013.19.1.30](http://dx.doi.org/10.26719/2013.19.1.30)

11. [Kumar GS, Varma S, Saenger MS, Burleson M, Kohrt BA, Cantey P. Noninfectious Disease Among the Bhutanese Refugee Population at a United States Urban Clinic. Journal of Immigrant and Minority Health. 2014. pp. 922–925. doi:](http://paperpile.com/b/QLrn6N/JUzVA)[10.1007/s10903-013-9800-1](http://dx.doi.org/10.1007/s10903-013-9800-1)

12. [Damiri B, Abualsoud MS, Samara AM, Salameh SK. Metabolic syndrome among overweight and obese adults in Palestinian refugee camps. Diabetol Metab Syndr. 2018;10: 34.](http://paperpile.com/b/QLrn6N/ztGgi)

13. [Bhatta MP, Shakya S, Assad L, Zullo MD. Chronic disease burden among Bhutanese refugee women aged 18–65 years resettled in Northeast Ohio, United States, 2008–2011. J Immigr Minor Health. 2015. Available:](http://paperpile.com/b/QLrn6N/4bnki) <https://idp.springer.com/authorize/casa?redirect_uri=https://link.springer.com/article/10.1007/s10903-014-0040-9&casa_token=gWF3pGCTXO0AAAAA:7v6NxrAIQ-6pWanhchraNkRTKN9Vs4OXxHosaawLXMGFq9l5nLBdXgPpk8uUrLamCNsFcr7LVxIHAhad_Q>

14. [Bhatta MP, Assad L, Shakya S. Socio-demographic and dietary factors associated with excess body weight and abdominal obesity among resettled Bhutanese refugee women in Northeast Ohio, United States. Int J Environ Res Public Health. 2014;11: 6639–6652.](http://paperpile.com/b/QLrn6N/ebftD)

15. [Hikichi H, Aida J, Kondo K, Tsuboya T, Kawachi I. Residential relocation and obesity after a natural disaster: A natural experiment from the 2011 Japan Earthquake and Tsunami. Sci Rep. 2019;9: 374.](http://paperpile.com/b/QLrn6N/hlYXs)

16. [Ohira T, Hosoya M, Yasumura S, Satoh H, Suzuki H, Sakai A, et al. Effect of Evacuation on Body Weight After the Great East Japan Earthquake. Am J Prev Med. 2016;50: 553–560.](http://paperpile.com/b/QLrn6N/oZtmh)

17. [Sakai A, Nakano H, Ohira T, Maeda M, Okazaki K, Takahashi A, et al. Relationship between the prevalence of polycythemia and factors observed in the mental health and lifestyle survey after the Great East Japan Earthquake. Medicine . 2020;99: e18486.](http://paperpile.com/b/QLrn6N/ECPWl)

18. [Nakamura K, Watanabe Y, Kitamura K, Kabasawa K, Someya T. Psychological distress as a risk factor for dementia after the 2004 Niigata-Chuetsu earthquake in Japan. J Affect Disord. 2019;259: 121–127.](http://paperpile.com/b/QLrn6N/tdZ32)

19. [Mulugeta W, Glick M, Min J, Xue H, Noe MF, Wang Y. Longitudinal Changes and High-Risk Subgroups for Obesity and Overweight/Obesity Among Refugees in Buffalo, NY, 2004–2014. Journal of Racial and Ethnic Health Disparities. 2018;5: 187–194.](http://paperpile.com/b/QLrn6N/cVOQt)

20. [Mulugeta W, Xue H, Glick M, Min J, Noe MF, Wang Y. Burden of Mental Illness and Non-communicable Diseases and Risk Factors for Mental Illness Among Refugees in Buffalo, NY, 2004-2014. J Racial Ethn Health Disparities. 2019;6: 56–63.](http://paperpile.com/b/QLrn6N/PwRjD)

21. [Bardenheier BH, Phares CR, Simpson D, Gregg E, Cho P, Benoit S, et al. Trends in Chronic Diseases Reported by Refugees Originating from Burma Resettling to the United States from Camps Versus Urban Areas During 2009-2016. J Immigr Minor Health. 2019;21: 246–256.](http://paperpile.com/b/QLrn6N/ARMM7)

22. [Nguyen M-LT, Rehkopf DH. Prevalence of Chronic Disease and Their Risk Factors Among Iranian, Ukrainian, Vietnamese Refugees in California, 2002-2011. J Immigr Minor Health. 2016;18: 1274–1283.](http://paperpile.com/b/QLrn6N/cQvOZ)

23. [Greene-Cramer B, Summers A, Lopes-Cardozo B, Husain F, Couture A, Bilukha O. Noncommunicable disease burden among conflict-affected adults in Ukraine: A cross-sectional study of prevalence, risk factors, and effect of conflict on severity of disease and access to care. PLoS One. 2020;15: e0231899.](http://paperpile.com/b/QLrn6N/xb3ro)

24. [Herrera-Fontana ME, Chisaguano AM, Villagomez V, Pozo L, Villar M, Castro N, et al. Food insecurity and malnutrition in vulnerable households with children under 5 years on the Ecuadorian coast: a post-earthquake analysis. Rural Remote Health. 2020;20: 5237.](http://paperpile.com/b/QLrn6N/kxNWj)

25. [Ebner DK, Ohsawa M, Igari K, Harada KH, Koizumi A. Lifestyle-related diseases following the evacuation after the Fukushima Daiichi nuclear power plant accident: a retrospective study of Kawauchi Village with long-term follow-up. BMJ Open. 2016;6: e011641.](http://paperpile.com/b/QLrn6N/A7TW6)

26. [Takahashi S, Yonekura Y, Tanno K, Shimoda H, Sakata K, Ogawa A, et al. Increase in Body Weight Following Residential Displacement: 5-year Follow-up After the 2011 Great East Japan Earthquake and Tsunami. J Epidemiol. 2021;31: 328–334.](http://paperpile.com/b/QLrn6N/Hxuka)
