# Supplementary material for: A systematic review of the burden of, access to services for and perceptions of patients with overweight and obesity, in humanitarian crisis settings
Source: PLoS One. 2023 Apr 24;18(4):e0282823. doi: 10.1371/journal.pone.0282823 (PMC10124894; doi:10.1371/journal.pone.0282823)
Supplement: S4 Appendix — (DOCX) [file pone.0282823.s005.docx]

## Appendix 4 - Details of Risk of Bias Assessment

|  | **external validity** | | | **internal validity** | | | | | |  |  |  |
| --- | --- | --- | --- | --- | --- | --- | --- | --- | --- | --- | --- | --- |
| **Author, year** | **A** | **B** | **C** | **D** | **E** | **F** | **G** | **H** | **I** | **external ROB** | **internal ROB** | **overall ROB** |
| Chandra et al., 2019 | No | No | No | Yes | Yes | Yes | Unclear | NA | No | High | Low | High |
| Yanni et al., 2013 | Yes | Yes | Yes | Yes | Yes | Yes | Yes | NA | Yes | Low | Low | Low |
| Bayyari et al., 2013 | No | Yes | Yes | Yes | Yes | Yes | Unclear | NA | Yes | Moderate | Low | Moderate |
| Drummond et al., 2011 | No | No | Yes | Yes | No | Yes | No | NA | Yes | High | High | High |
| Bhatta et al., 2015 (1) | No | No | Yes | Yes | Yes | Yes | Yes | NA | No | High | Moderate | High |
| Bhatta et al., 2014 (2) | No | No | Unclear | Yes | Yes | Yes | Yes | NA | Unclear | High | Moderate | High |
| El Kishawi et al., 2014 | No | Yes | Yes | Yes | Yes | Yes | Yes | NA | Unclear | Moderate | Low | Moderate |
| Furusawa et al., 2011 | No | Yes | No | Yes | Yes | Yes | Yes | NA | Yes | High | Low | High |
| Kory et al., 2013 | No | Yes | Unclear | Yes | Yes | Yes | Unclear | Na | Unclear | High | Moderate | High |
| Mulugeta et al, 2018 (1) | No | No | No | Yes | Yes | Yes | Unclear | Na | Yes | High | Moderate | High |
| Mulugeta et al. (2) 2019 | No | No | No | Yes | Yes | Yes | Unclear | Na | Yes | High | Moderate | High |
| Mulugeta et al. (3) 2019 | No | No | No | Yes | Yes | Yes | Unclear | Na | Yes | High | Moderate | High |
| Sakai et al. 2020 | Yes | Yes | No | Yes | No | Yes | Yes | Yes | Yes | High | Low | High |
| Ohira, 2016 | No | Yes | No | Yes | Yes | Yes | Yes | NA | No | High | Moderate | High |
| Ohira et al.., 2017 | No | Yes | No | Yes | Yes | Yes | Yes | NA | No | High | Moderate | High |
| Satoh et al., 2021 | No | No | No | No | No | No | No | N/A | No | High | High | High |
| Dhair et al., 2020 | No | No | No | Yes | No | No | No | NA | Yes | High | High | High |
| Kim et al.., 2018 | No | No | No | Yes | Yes | Yes | Yes | NA | Yes | High | Low | High |
| Kim et al., 2015 | No | No | No | Yes | Yes | Yes | Yes | NA | No | High | Low | High |
| Jung Kim et al., 2016 | No | No | No | Yes | No | Yes | Yes | NA | No | High | Moderate | High |
| Jeong et al. | No | No | No | Yes | Yes | Yes | Yes | Unclear | Yes | High | Low | High |
| Renzaho et al., 2014 | No | No | No | Yes | Yes | Yes | Yes | NA | Yes | High | Low | High |
| Abdollahi et al., 2015 | No | No | No | Yes | Yes | Yes | Yes | NA | No | High | High | High |
| Kumar et al., 2014 | No | No | Unclear | Unclear | Yes | Unclear | Unclear | NA | Yes | High | High | High |
| Reznar et al., 2020 | No | Yes | Unclear | Yes | Yes | Unclear | Unclear | NA | No | High | High | High |
| Taherifard et al., 2021 | No | Yes | Yes | Yes | Yes | Yes | Yes | NA | Yes | High | Low | High |
| Sastre L, 2020 | No | No | Yes | Yes | Yes | Yes | Yes | NA | No | High | Low | High |
| Amstutz et al., 2020 | No | No | No | Yes | Yes | Yes | Yes | NA | Yes | High | Low | High |
| Jen et al., 2015 (1) | No | Yes | Yes | Yes | Yes | No | No | NA | No | High | High | High |
| Jen et al., 2018 (2) | No | No | Yes | Yes | Yes | No | No | NA | No | High | High | High |
| Rhodes et al., 2016 | No | No | No | Unclear | Yes | Unclear | Unclear | NA | No | High | High | High |
| Greene-Cramer et al., 2020 | No | Yes | No | Yes | Yes | Yes | No | NA | Yes | High | High | High |
| Herrera-Fontana et al., 2019 | No | No | Yes | Yes | Yes | Yes | Unclear | NA | Yes | High | High | High |
| Mansour et al., 2020 | Yes | Yes | No | Yes | Yes | Yes | Yes | NA | No | Moderate | Low | Moderate |
| Modesti et al., 2020 | No | No | Yes | Yes | Yes | Yes | Yes | NA | Yes | High | Low | High |
| Ratnayake et al., 2020 | No | Yes | Yes | Yes | Yes | Yes | Yes | NA | No | Moderate | Low | Moderate |
| Takahashi et al., 2020 (1) | Yes | No | No | Yes | No | Yes | Yes | N/A | Yes | High | Moderate | High |
| Takahashi et al., 2016 (2) | Yes | Yes | No | Yes | No | Yes | Yes | NA | Yes | High | Moderate | High |
| Takahashi et al., 2020, (3) | Yes | No | No | Yes | Yes | Yes | Yes | N/A | Yes | High | Moderate | High |
| Eryurt & Menet, 2019 | Yes | Yes | No | Yes | Yes | Yes | Yes | NA | Yes | Moderate | Low | Moderate |
| Hikichi et al., 2019 | No | Yes | No | Yes | Yes | Yes | Unclear | NA | Yes | High | High | High |
| Maldari et al., 2019 | No | Yes | Yes | Yes | Yes | Yes | Yes | NA | No | High | Moderate | High |
| al.-Duais et al., 2019 | No | Yes | Yes | Yes | Yes | Yes | Yes | NA | Yes | High | High | High |
| Amr et al., 2019 | No | No | Yes | Yes | Yes | Yes | Yes | NA | Yes | High | Low | Moderate |
| Bardenheier et al., 2019 | Yes | Yes | Yes | Yes | Yes | Yes | Yes | NA | Yes | Low | Low | Low |
| Damiri et al., 2019 | No | Yes | Yes | Yes | Unclear | Yes | Yes | NA | No | High | Moderate | High |
| Nakamura et al., 2019 | No | No | Yes | Yes | Unclear | Yes | Yes | Yes | Yes | High | Moderate | High |
| Kortas et al.., 2017 | No | Yes | No | Yes | Yes | Yes | Yes | NA | Yes | High | Low | Moderate |
| Adrega et al.., 2018 | Yes | Unclear | Yes | Yes | No | Yes | Yes | NA | No | Moderate | High | Moderate |
| Damiri et al.., 2018 | No | Yes | Yes | Yes | Yes | Yes | Yes | NA | Yes | Moderate | Low | Moderate |
| Naigaga et al.., 2018 | Yes | Yes | Unclear | Yes | Yes | Yes | Yes | NA | Yes | Low | Low | Low |
| Bardenheier et al.., 2018 | Yes | Yes | Yes | Yes | Yes | Yes | Yes | NA | Yes | Low | Low | Low |
| Singh et al., 2015 | No | No | No | Yes | Yes | Yes | Yes | NA | Yes | High | Low | High |
| Balcilar, 2016 | Yes | Yes | Yes | Yes | Yes | Yes | Yes | NA | Yes | Low | Low | Low |
| Ebner et al., 2016 | No | No | No | Yes | Yes | Yes | Unclear | NA | No | High | Low | High |
| Nguyen et al., 2015 | Yes | Yes | Yes | Yes | Yes | Yes | Yes | NA | Yes | Low | Low | Low |

A= Was the sampling frame a true or close representation of the target population?

B= Was some form of random selection used to select the sample, or was a census undertaken?

C= Was the likelihood of non-response bias minimal?

D= Were data collected directly from the subjects (as opposed to a proxy)?

E= Was an acceptable case definition used?

F= Was the study instrument that measured the parameter of interest shown to have reliability and validity?

G= Was the same mode of data collection used for all subjects?

H= Was the length of the shortest prevalence period for the parameter of interest appropriate?

I= Were the numerator and denominator for the parameter of interest appropriate?
