# Supplementary material for: A systematic review of the burden of, access to services for and perceptions of patients with overweight and obesity, in humanitarian crisis settings
Source: PLoS One. 2023 Apr 24;18(4):e0282823. doi: 10.1371/journal.pone.0282823 (PMC10124894; doi:10.1371/journal.pone.0282823)
Supplement: Attachment — Submitted filename: response to reviewers - 23.02.23.docx [file pone.0282823.s007.docx]

Dear Che and team,

Re: PONE-D-22-11727R1

A systematic review of the burden of, access to services for and perceptions of patients with overweight and obesity in humanitarian crisis settings

Thank you taking the time to review the revision of our paper. We are pleased that the reviewer feels that all previous points have been addressed. Further response below:

I noticed that there may be minor errors in the reference order.  It looks as if references 104 and 105 (page 38) now comes before reference 103 (page 39) in the updated version. Similarly references 107-109 come before 106 in the revised version. Please review and correct references where needed. Thank you.

Thank you for raising this point. We have checked and updated all the references, and these are now in the correct order.

Thank you once again

Farah Kidy
